# Emotion Regulation Interventions for Cancer Patients and Their Relatives: A Systematic Review

**DOI:** 10.1002/cam4.71514

**Published:** 2026-02-12

**Authors:** Ambre Naeyaert, Valentyn Fournier, Laura Caton, Marie‐Charlotte Gandolphe, Christelle Duprez, Lisa Laroussi‐Libeault, Marie Vander Haegen, Pascal Antoine, Kristopher Lamore

**Affiliations:** ^1^ Université de Lille, CNRS, UMR 9193—SCALab—Sciences Cognitives et Sciences Affectives Lille France; ^2^ Maisonneuve‐Rosemont Hospital Research Center, Montreal Quebec Canada; ^3^ Department of Anesthesiology and Pain Medicine, Faculty of Medicine Université de Montréal Quebec Canada; ^4^ Division of Hematology, Oncology and Cellular Therapy, Hôpital Maisonneuve‐Rosemont, Montreal Quebec Canada; ^5^ Université de Liège Laboratoire RUCHE, Research Unit for a Life‐Course Perspective on Health and Education Liège Belgium

**Keywords:** emotion regulation, intervention, oncology, patients, relatives, systematic review

## Abstract

**Background:**

Emotional regulation (ER) is crucial for the psychological well‐being of patients with cancer and their relatives. Various ER interventions exist but often suffer from poor adherence and high attrition rates.

**Aims:**

This systematic review aims to identify ER interventions for patients with cancer and their relatives, describe their content, development, and evaluation methods, and assess their efficacy on ER and psychological health outcomes.

**Methods:**

Cochrane, Embase, PsycArticles, PsycINFO, and Web of Science databases were searched. Eligible studies examined individual interventions targeting ER (Intervention) in adult patients with cancer and their relatives (Population), included a control group or a pre–post assessment (Comparator), and reported outcomes related to ER or psychological health outcomes using quantitative or mixed‐method results (Study type).

**Results:**

Eight studies were included. Most interventions were theoretically grounded in cognitive behavioral therapy (CBT) and followed a similar structure: informational sessions, skill‐building modules using targeted therapeutic techniques, and consolidation via homework and relapse‐prevention exercises. Despite high attrition rates, most interventions significantly improved ER and psychological outcomes. Some trials lacked statistical significance, mainly due to methodological limitations. Although acceptability was rarely assessed, evidence suggests patient–relatives dyadic approaches may better meet participant needs.

**Conclusion:**

ER interventions share common features and show effectiveness but sometimes lack alignment between objectives and evaluated outcomes. Retention remains a major challenge, emphasizing the need to redesign interventions and their implementation. Future research should explore attrition causes and develop strategies to enhance engagement.

## 1 Introduction

Patients with cancer and their relatives experience significant psychological distress [1, 2], which is partly due to difficulties with emotion regulation (ER) [3], that negatively affects their quality of life [4]. The prevalence of psychological distress is estimated to be 43%–64.5% among cancer patients [5, 6] and 41%–96% among the caregivers of cancer patients [7, 8]. ER is defined as the processes involved in monitoring, evaluating, and changing emotional responses [9]. In a cancer context, ER is shaped by a complex interplay among cancer‐specific, psychological, and demographic factors. High levels of cancer‐related fear [10] and the use of maladaptive strategies such as expressive suppression, avoidance, rumination, catastrophizing, and self‐blame can disrupt emotional processing [3, 11]. For example, body image disturbances among breast cancer patients, especially following visible physical changes like mastectomy or hair loss, can evoke shame, fear, or avoidance and impair emotional functioning [12]. In addition, demographic and individual characteristics play a role in ER: alexithymia (i.e., difficulties with identifying and describing emotions) [13], a younger age [14], and a family history of cancer [15] are associated with ER difficulties. Therefore, ER has been recognized as a transdiagnostic process underlying numerous psychological symptoms [16], making ER a promising target for interventions focused on symptom management and the prevention of psychopathology via the enhancement of adaptive ER capacities such as cognitive reappraisal or acceptance, which are linked to better mental health and quality of life [3].

Given the link between ER and emotional distress [3, 17], ER‐focused interventions have been developed. ER interventions shown efficacy in improving adaptive ER capacities in patients with cancer, even after 12 months [18]. A*daptive ER capacities* such as cognitive reappraisal, acceptance, problem‐solving, and mindfulness support psychological well‐being and long‐term adjustment [19]. In contrast, *maladaptive capacities*, such as suppression, rumination, or avoidance, are associated with increased distress [20]. Thus, ER plays a central role in psychological adjustment to various scenarios, including chronic illness and cancer care [3, 11]. For instance, suppression (inhibiting the expression of negative emotions) is associated with higher distress; cognitive reappraisal (reinterpreting situations to reduce their emotional impact) may alleviate it [8].

Beyond patients with cancer, the ER capacity of their relatives is also important. In non‐cancer populations, dyadic ER support couple satisfaction and individual psychological well‐being [21]. This relevance in cancer context has sparked interest in tailored interventions [22]. Emotion regulation therapy (ERT), a cognitive behavioral therapy (CBT) focusing on emotional processes [23], has shown promise in reducing negative emotions [24], anxio‐depressive symptoms, and ER difficulties among relatives of patients with cancer [23]. Furthermore, group interventions appear especially beneficial for both patients and their relatives [22].

Recommendations for developing non‐pharmacological interventions [25, 26] highlight the need to clearly define target outcomes and the mechanisms through which interventions operate. Although interventions addressing emotional outcomes in patients with cancer and their relatives exist, their development and evaluation are often unclear, and their formats vary widely. Additionally, the conceptualization of ER varies across studies [27], creating confusion about what is being targeted. Given the prevalence of unmet emotional needs (i.e., a lack of psychological support in coping with stress [28, 29]) a synthesis of interventions targeting ER is needed to clarify which elements may influence their efficacy. To our knowledge, there is currently no standardized framework dedicated to the classification of ER intervention components, unlike behavior change interventions, which benefit from structured taxonomies such as Behavior Change Techniques [30]. The aims of this systematic review are, therefore, to (1) identify existing interventions targeting ER in patients with cancer and their relatives, (2) describe their content and developmental/evaluative methodological frameworks, and (3) describe their efficacy in terms of ER and psychological health outcomes (e.g., distress, anxiety, depression).

## 2 Methods

This study was conducted and reported in accordance with the Preferred Reporting Items for Systematic Reviews and Meta‐Analysis Statement (PRISMA) guidelines [31]. The review protocol was pre‐registered in PROSPERO (https://www.crd.york.ac.uk/PROSPERO/view/CRD42023484110).

### 2.1 Eligibility Criteria

We included studies on interventions targeting ER in adult patients with cancer or their relatives. Our review focused on individual interventions for one member of the cancer dyad, as dyadic programs are rare, heterogeneous, and often only partially address ER. This allowed comparison of coherent intrapersonal ER protocols and reduced methodological variability. Eligible studies had a control group or pre–post measures and reported ER or psychological outcomes (e.g., anxiety, depression, quality of life). Only interventional studies with quantitative or mixed methods (RCTs, non‐RCTs, pre–post designs) were included. We excluded pediatric, end‐of‐life/palliative care studies, and those lacking quantitative data or comparison groups.

### 2.2 Information Sources

We searched the major electronic databases (i.e., CINAHL, Cochrane, Embase, PsycArticles, PsycINFO, PubMed, and Web of Science) on November 22, 2023 (updated on October 31, 2024). Gray literature was sought via Google Scholar and Google Web Search. Finally, reference lists of included studies were manually checked for completeness.

### 2.3 Search Strategy

When applicable, the search was restricted to peer‐reviewed articles reporting on human studies that were written in English, French, or Spanish. No date restriction was applied. The full search strategy is presented in Appendix A.

### 2.4 Selection Process

We used the web application Covidence (www.covidence.org) for study selection. Following the automatic removal of all duplicates, titles and abstracts were independently screened by six researchers (A.N., L.C., V.F., M.V.H., K.L.). Full texts were then independently screened by four researchers (A.N., L.C., V.F., K.L.). Any disagreements between the researchers were resolved by screeners reaching a consensus.

### 2.5 Data Collection Process

The research team discussed and defined the elements to be extracted from the included studies. These elements were then incorporated into a template used by three researchers (V.F., A.N., and L.C.) for data extraction. A.N. performed a global check for each study.

### 2.6 Data Items

The following data were extracted: participant characteristics (age, disease site and stage, treatment, and baseline psychological measurements); intervention characteristics (intervention type, aim, intervention developmental framework, intervention duration, mode of delivery, study duration, main targets, and measurements); intervention content (information, therapeutic processes, adaptive capacities, between‐sessions tasks, and relapse prevention); and intervention effects (pre–post measurements, control group comparisons, change scores, statistical significance (*p* values), and effect sizes).

### 2.7 Risk of Bias Assessment

We assessed the risk of bias using different tools for RCTs and non‐RCTs. For RCTs, we used the RoB 2 tool [32], which is recommended by Cochrane reviews. This tool identifies five domains of bias: (1) bias arising from the randomization process, (2) bias due to deviations from intended interventions, (3) bias due to missing outcome data, (4) bias in the measurement of outcomes, and (5) bias in the selection of the reported results. We used the ROBINS‐I (Risk of Bias In Non‐randomized Studies of Interventions) tool to evaluate non‐RCTs [33]. ROBINS‐I examines seven domains of bias: (1) bias due to confounding, (2) bias in the selection of study participants, (3) bias in the classification of interventions, (4) bias due to deviations from intended interventions, (5) bias due to missing data, (6) bias in the measurement of outcomes, and (7) bias in the selection of the reported results. Two researchers (V.F., A.N.) independently assessed the methodological quality of each study. V.F. and A.N. resolved any disagreements through discussion to reach a consensus.

### 2.8 Synthesis Methods

We performed a narrative synthesis to describe interventions targeting the ER of patients with cancer or their relatives, including their content and the methodological framework for their development and intervention (where applicable). The global efficacy of the interventions was described and assessed by collecting relevant information (e.g., statistical significance, effect sizes, attrition rate) and performing a narrative synthesis of those results. Given the heterogeneity in interventions and outcomes, we adopted a narrative synthesis approach, in accordance with Synthesis Without Meta‐analysis (SWiM) guidelines [34], which provide a structured framework for synthesizing findings when a meta‐analysis is not appropriate. To meet our objectives and following structured data extraction (see Section 2.6), we compared studies based on their shared design, content, and measured effect components. This allowed us to identify common patterns and evaluate overall efficacy, despite variability in methodologies and reported outcomes.

### 2.9 Certainty of Evidence

A systematic Grading of Recommendations Assessment, Development, and Evaluation (GRADE) assessment [35] was conducted to determine the certainty of evidence from the included studies. The full assessment covered six domains: risk of bias, imprecision, inconsistency, indirectness, effect size, and publication bias. We classified outcomes as either critical, referring to those directly related to ER or its core components (e.g., mindfulness, experiential avoidance, acceptance), or important, including outcomes such as psychological distress, anxiety, depression, and fear of cancer recurrence. Interventions were evaluated separately for each outcome because there were substantial differences in their content, mode of delivery, and comparators. Even when several studies examined the same outcome, typically, only one study contributed to the evaluation of each specific intervention. This approach allowed us to provide intervention‐specific, GRADE‐based recommendations, rather than pooling heterogeneous interventions that were not directly comparable.

## 3 Results

### 3.1 Study Selection

**FIGURE 1:**
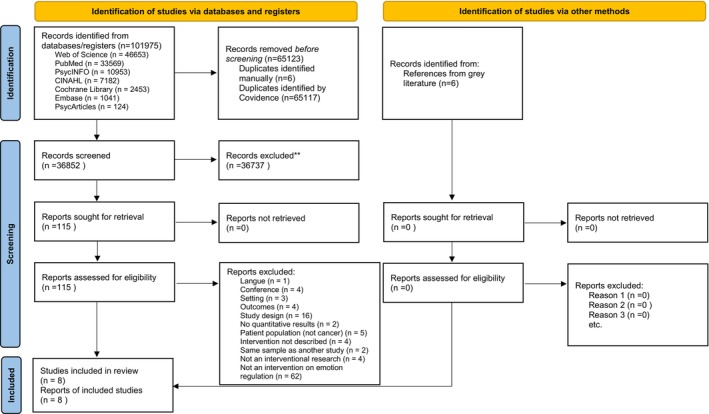
PRISMA flowchart of the study selection process. *Consider, if feasible to do so, reporting the number of records identified from each database or register searched (rather than the total number across all databases/registers). **If automation tools were used, indicate how many records were excluded by a human and how many were excluded by automation tools. *Source:* Page et al. [31]. This work is licensed under CC BY 4.0. To view a copy of this license, visit https://creativecommons.org/licenses/by/4.0/.

Of the 101,975 studies retrieved from the databases, 65,123 duplicates (65,117 identified by Covidence and six manually) were removed, resulting in 36,852 studies for title and abstract screening. During this phase, 320 conflicts were identified among reviewers, resulting in an inter‐rater agreement of approximately 99.13% (= 36,532/36,852). The inter‐rater reliability (Cohen's Kappa) across reviewer pairs ranged from 0.14 to 0.67 (see Appendix B), reflecting variations in screening experience and an imbalance in the distribution of inclusion criteria. The discrepancy between the very high percentage agreement and the lower kappa values is explained by an unbalanced distribution of categories, a well‐known phenomenon that reduces kappa despite high observed agreement. Following this, the full text of 115 studies was reviewed to assess their eligibility. Subsequently, 107 studies were excluded, resulting in eight studies for inclusion in the review. Figure 1 is a flowchart detailing the reasons for the exclusion of studies.

### 3.2 Quality Assessment of Included Studies

Table 1 contains a summary of the quality assessment of the studies.

**TABLE 1:** Appraisal of the quality of studies.

ROB (RCT studies)						
	Domain 1: Risk of bias arising from the randomization process	Domain 2: Risk of bias due to deviations from the intended interventions (effect of assignment to intervention) + (effect of adhering to intervention)	Domain 3: Missing outcome data	Domain 4: Risk of bias in measurement of the outcome	Domain 5: Risk of bias in selection of the reported result	Overall risk of bias
Thilges et al. (2023)	●	● ●	●	●	●	●
Jacobsen et al. (2002)	●	● ●	●	●	●	●
Nápoles et al. (2015)	●	● ●	●	●	●	●
O'Toole et al. (2020)	●	● ●	●	●	●	●
Smith et al. (2023)	●	● ●	●	●	●	●
Farnoodimehr et al. (2021)	●	● ●	●	●	●	●

ROBIN (pre‐post studies NOT RCT)								
	Bias due to confounding & questions relating to baseline and time‐varying confounding	Bias in selection of participants into the study	Bias in classification of interventions	Bias due to deviations from intended interventions	Bias due to missing data	Bias in measurement of outcomes	Bias in selection of the report result	Overall bias risk of bias judgment
Almeida et al. (2022)	●	●	●	●	●	●	●	●
Applebaum et al. (2020)	●	●	●	●	●	●	●	●

*Note:* ● = Low risk, ● = Some concerns, ● = High risk.

The two non‐RCT studies (pre–post designs) were judged to have a moderate overall risk of bias [23, 36], with seven domains rated low risk and nine moderate. Of the RCT studies (*n* = 6), only one fully met the RoB‐2 criteria, indicating a low risk of bias [37]. Three studies partially met domain 1 [38, 39, 40] and three others were judged as having a low risk in this domain [37, 40, 41]. For domain 2 (effect of assignment), three partially met criteria [38, 40, 41], and two had no apparent risk [37, 40]. Regarding adherence, three studies were rated partial [38, 39, 41], and two were rated high risk [40, 42]. Only one [40] showed high risk in domain 3. For domain 4, studies were either at low [37, 39, 41, 42] or moderate [38, 40] risk. All studies showed a low risk for domain 5. Overall, three RCTs were moderate risk [27, 28, 31] two low [26, 30], and one high [29].

### 3.3 Study Characteristics

The included studies, published between 2002 and 2024, were conducted in various countries, including Portugal [35], the USA [23, 37, 38, 41] Iran [39], and Denmark [42], as well as in Australia, New Zealand, the UK, and Canada [40].

The majority of studies (*n* = 6) were RCTs [37, 38, 39, 40, 41, 42], while two employed pre–post designs [23, 35]. All studies reported quantitative data, with two also including qualitative findings [16, 25]. Control groups for RCT interventions included a waitlist [42] usual care [37, 38, 41], a healthy lifestyle active control intervention (CanCope Lifestyle; CL [40]), or medication [39]. There were six single‐center [23, 35, 38, 39, 41, 42] and two multi‐center studies [36, 40]. Study durations ranged from 2 months to 2 years.

### 3.4 Participant Characteristics

A summary of the participants' characteristics is presented in Table 2.

**TABLE 2:** Participants' characteristics of the included studies.

Authors	Age (mean (SD))	Cancer site	Stage of the disease (%/mean (SD))	Stage throughout cancer journey (%/mean (SD))	Measurement at baseline	Psychological states at baseline (%/mean (±SD))
Almeida et al. (2022)	45 (11.25)	Hematological, colorectal, gynecological, urologic, breast	0/I (29%); II (47%); III/IV (24%)	No treatment (76%) On treatment (24%)	Personal Questionnaire (PQ) Changes in psychological difficulties during therapy—Fear of Recurrence (FCR) Psychological Distress The Clinical Outcome in Routine Evaluation (CORE‐OM)	Major FCR (53%) Minor FCR (47%) Distress: Mean (M) = 18.38, SD = 5.15
Applebaum et al. (2020)	54.45 (11.14)	NA (caregivers)	NA	NA	Anxiety and Depression Scale (HADS) The Difficulties in Emotion Regulation Scale (DERS)	Anxiety and Depression 18.87 (16.51, 21.24) Difficulties in emotion regulation 81.94 (74.32, 89.55)
Jacobsen et al. (2002)	56 (12)	Breast, lung, ovarian, lymphoma	NS	Chemotherapy treatment (58%)	Center for Epidemiologic Studies Depression Scale (CES‐D) State–Trait Anxiety Inventory Scale (STAI‐S)	NS
Nápoles et al. (2015)	50.5 (10.9)	Breast	0 = 20 (26); I = 12 (16) II = 31 (41); III = 13 (17)	Surgery (breast conserving: 43 (57); mastectomy: 33 (43)) Adjuvant treatment (both chemotherapy and radiation: 33 (43), only radiation: 18 (24); only chemotherapy: 14 (18)) No treatment: 11 (15)	The Functional Assessment of Cancer Therapy‐Breast (FACT‐B)	Breast cancer‐specific quality of life—Emotional well‐being (12.07, 64.91)
O'Toole et al. (2020)	Caregivers: 49.4 (15.2) Patients: 60.2 (7.8)	Colon, lung, rectal, ventricular, pancreatic, bile duct, esophagus, ovary, uterus, sarcoma	II = 1 (4); III = 6 (20); IV = 18 (62)	NA	Distress Thermometer Hospital Anxiety and Depression Scale (HADS) Difficulties in Emotion Regulation Scale (DERS)	Caregivers' outcomes Anxiety & Depression: 20.5 (7.1). Difficulties with emotion regulation: 86.9 (20.7) Patients' outcomes Anxiety & Depression: 12.1 (6.3)
Smith et al. (2023)	NS	NA (cancer survivors)	NA	Remission	Mini International Neuropsychiatric Interview (MINI)	Generalized Anxiety Disorder CanCopeMind (30%) Major Depression CanCopeMind (22%)
Thilges et al. (2023)	NS	Head and neck	NS	Diagnosis (not yet initiated treatment)	Beck Depression Inventory‐II (BDI‐II) Brief Symptom Inventory‐18 (BSI‐18)	NS
Farnoodimehr et al. (2021)	NS	Breast	6‐month illness	NS	The Depression, Anxiety, Stress Scale (DASS)	Depression: 13.05 (1.73) Anxiety: 14.22 (1.47) Stress: 19.61 (2.19)

Abbreviations: NA, not applicable; NS, not specified; SD, standard deviation.

The interventions were administered to all types of patients with cancer, including those in the active phase of the disease [23, 35, 37, 38, 39, 41, 42] those undergoing active treatments (i.e., during the intervention phase) [23, 35, 37, 38, 41], and those in remission [40] or recently diagnosed without active treatment [39]. At baseline, participants frequently exhibited high levels of psychological distress, including anxiety and depression symptoms, and these outcomes served as inclusion criteria in some studies [23, 35, 39].

### 3.5 Intervention Characteristics

A summary of the intervention characteristics is presented in Table 3.

**TABLE 3:** Interventions characteristics.

Authors	Type of intervention	Aims	Conceptual framework	Mode of delivery	Length	Duration of sessions (mean (SD))	Main targets	Measurement
Almeida et al. (2022)	EFT‐CA	To evaluate the efficacy of EFT as a potentially effective treatment for fear of cancer recurrence	Emotion Focused Therapy	Therapist	Between 4 weeks to 16 weeks	13 sessions (6.26)	Stress management	Fear of recurrence (PQ) Psychological distress (CORE‐OM)
Applebaum et al. (2020)	ERT‐C	To evaluate the acceptability and initial efficacy of ERT adapted to the experience of cancer ICs (ERT‐C) on distress and perseverative negative thinking (PNT)	Translational Affect Science	Therapist	8 weeks	8 weekly sessions lasting 1 h	Emotional regulation	Perseverative Negative Thinking: Rumination (RRS) and Worry (PSWQ), Distress (DT), Caregiver burden (CRA), Depression and Anxiety (HADS), Dysregulation of emotion (DERS), Mindfulness (FFMQ)
Jacobsen et al. (2002)	Non‐specific psychosocial interventions (e.g., PSMT and SSMT)	To improve access to psychosocial interventions during chemotherapy treatment by evaluating the efficacy and costs of a patient self‐administered form of stress management training that requires limited professional time or experience to deliver	Cognitive behavioral theory	Therapist (PSMT) booklet and audiotape self‐guided (SSMT)	NS	4 times corresponding to the chemotherapy cycle. 65 min (SSMT condition) 60 min (PSMT condition)	Stress management	Quality of Life (SF‐36), Depression (CES‐D), Anxiety (STAI‐S)
Nápoles et al. (2015)	Non‐specific psychosocial intervention	To assess a community‐based, translational stress management program to improve health‐related quality of life in Spanish‐speaking Latinas with breast cancer.	Cognitive behavioral theory	Patients partners (“compa ñeras”)	8 weeks	8 weekly sessions 90 min	Stress management (e.g., self‐efficacy, coping skills, perceived social support)	Breast cancer specific quality of life (FACT‐B), General Distress Symptom (BSI) (e.g., depression, anxiety, somatization), breast‐cancer specific distress (subscale ITS of IES)
O'Toole et al. (2020)	ERT‐C	To evaluate the efficacy of Emotion Regulation Therapy adapted for caregivers (ERT‐C) on psychological and inflammatory outcomes in psychologically distressed ICs and the cancer patients cared for.	Translational Affect Science	Master's or doctorate‐level students supervised by the first author	8 weeks	8 weekly sessions lasting 60 min	Emotional regulation	Psychological distress (DT), Anxiety and Depression (HADS). Perseverative Negative Thinking: Rumination (RRS‐B) and Worry (PSWQ), Caregiver Burden (CRA), Quality of Life (WHO‐5), Dysregulation of emotion (DERS), Mindfulness (FFMQ), Decentering (EQ), cognitive reappraisal (subscale of ERQ), Quality of life (QLQ‐C30)
Smith et al. (2023)	CBI (UP)	To assess the efficacy of an emotional‐focused, modular, Internet‐delivered adaptation of the Unified Protocol (UP) in improving cancer survivors' emotion regulation strategies	Unified Protocol (UP) Cognitive behavioral therapy and trans‐diagnostic treatment	Automatized self‐guided online format (e.g., laptop, computer, smartphone, tablet)	8 weeks	8 weeks 1 h weekly	Stress management	Negative beliefs about emotions (BES), Mindfulness (SMQ), Cognitive reappraisal skills (UP‐CSQ), Emotional regulation (CERQ): Catastrophizing (CERQ‐C) and Refocus on Planning (CERQ‐R), Experiential avoidance (MEAQ‐30)
Thilges et al. (2023)	CBI	To evaluate a brief, seven‐session cognitive behavioral intervention (BCI) on self‐efficacy for coping with cancer, depression and quality of life in patients receiving treatment for squamous cell carcinoma of the head and neck (HNC)	Cognitive behavioral theory	Therapist	12 weeks	7 sessions in‐person lasting 45‐50 min + 1 session phone lasting 20 min	Stress management (e.g., self efficacy)	Depression (BDI‐II), Somatization, depression and anxiety (BSI‐18), Quality of Life (FACT‐H&N), self‐efficacy for coping with cancer (CaBI)
Farnoodimehr et al. (2021)	CBI (UP)	To evaluate the effectiveness of Unified Protocol (UP) on emotional problems of women who suffer from breast cancer.	Unified Protocol (UP) Cognitive behavioral therapy and trans‐diagnostic treatment	Trained Master's student in Clinical Psychology and Cognitive‐Behavioral Therapy	8 weeks	12 sessions lasting 60 min of the UP weekly	Stress management	Depression, anxiety and Stress (DASS), Emotional Regulation (ERQ), Psychological flexibility (AAQ‐II)

*Note:* NS: The study does not explicitly specify the exact duration of the intervention in months, but given that follow‐up assessments occurred before the 2nd, 3rd, and 4th chemotherapy cycles.

Abbreviations: Questionnaires: AAQ‐II, Acceptance and Action Questionnaire; BDI‐II, Beck Depression Inventory‐II; BES, Beliefs about Emotion Scale; BSI, Brief Symptom Inventory; BSI‐18, Brief Symptom Inventory‐18; CaBI, Cancer Behavioral Inventory‐Brief Form; CBI, Cognitive Behavioral Intervention; CERQ‐C, Catastrophizing; CERQ‐R, Refocus on Planning; CES‐D, Center for Epidemiologic Studies Depression Scale; CORE‐OM, Clinical Outcome in Routine Evaluation; CRA, Caregiver Reaction Assessment; DASS, Depression, Anxiety, Stress Scale; DERS, Difficulties in Emotion Regulation Scale; DT, Distress Thermometer; EFT‐CA, Emotional Freedom Technique for Caregivers; EQ, Experiences Questionnaire; ERT‐C, Emotional Regulation Training for Caregivers; FACT‐B, Functional Assessment of Cancer Therapy‐Breast; FACT‐H&N, Functional Assessment of Cancer Therapy‐Head and Neck Version; FFMQ, Five Facet Mindfulness Questionnaire; HADS, Hospital Anxiety Depression Scale; IES, Intrusive Events Scale; ITS, Intrusive Thoughts Scale; MEAQ‐30, Multidimensional Experiential Avoidance Questionnaire‐30; PQ, Personal Questionnaire; PSWQ, Penn State Worry Questionnaire; QLQ‐C30, Cancer Core Quality of Life Questionnaire; RRS, Rumination Response Scale; RRS‐B, Rumination Response Scale—Brooding subscale; SF‐36, Medical Outcomes Study 36‐Item Short Form; SMQ, Southampton Mindfulness Questionnaire; STAI‐S, State–Trait Anxiety Inventory Scale; UP, Unified Protocol; UP‐CSQ, Unified Protocol‐Cognitive Skills Questionnaire; WHO‐5, World Health Organization Questionnaire. Other abbreviations: PSMT, professionally administered stress management training; SSMT, self‐administered stress management training.

All studies aimed to assess effects on ER (e.g., psychological flexibility, reappraisal), emotional symptoms (e.g., anxiety, depression, fear of recurrence), and quality of life. Only two assessed acceptability [23, 35], and none reported ecological assessments. Most interventions targeted patients with cancer; only two focused on relatives [23, 42]. All interventions were delivered face‐to‐face, except for one self‐guided online program [40]. Session frequency ranged from 4 [38] to 16 [35], with most offering eight weekly sessions [23, 37, 42]. In the self‐guided online intervention [40], the sessions could be completed in as few as 2 weeks. One study states that the intervention took place over a 12‐week period, with sessions scheduled at 2‐week intervals and a final session in week 12 [41]. Intervention durations varied, with most sessions lasting from approximately 45 [41] to 60 min [23, 35, 39, 40, 41, 42], while some lasted up to 90 min [36]. One study linked sessions to chemotherapy cycles [38].

Follow‐up periods varied, ranging from 2 [39] to 12 months [41], with 3‐month [37, 40, 41, 42] and 6‐month [36, 41] follow‐ups being the most common. One study tailored delivery to individual needs (e.g., proximity, financial or physical constraints) [35]. Additionally, CBT was adapted into two caregiver‐focused programs: ER training (ERT‐C) [23] and the Emotion‐Focused Therapy (EFT‐CA) [35].

### 3.6 Intervention Activity Content

A summary of each intervention's content is presented in Table 4.

**TABLE 4:** Content of the interventions.

Authors	Interventions	Information	Therapeutic processes	Adaptive capacities (skills and strategies)	Consolidation (between‐session tasks and relapse prevention)
Almeida et al. (2022)	EFT‐CA	NS	Two‐Chair Work, Experiential Focusing, Trauma Retelling	Identification of emotion Meaning‐Making Exploring Emotional Needs Emotions activation (expression) Working with parts of self Self‐compassion Insight/Awareness	NS
Applebaum et al. (2020)	ERT‐C	Introduction to ERT‐C (e.g., cue detection/self‐monitoring) (session 1)	Information on intervention (e.g., cue detection and self‐monitoring) (Session 1) Attention regulation (Session 2) Distancing, reappraisal, and compassionate reframing (Session 3) On‐the‐spot regulation techniques (Session 4) Imaginal and in vivo exposure (Sessions 5–7)	Emotional and motivational awareness (Session 1) Attentional regulation during distress (Session 2) Metacognitive emotion regulation (Session 3) In‐the‐moment regulatory responding (Session 4) Behavioral activation aligned with personal values (Sessions 5–7)	Review of skills: Using skills autonomously in daily life situations (Session 8)
Jocabsen et al.(2002)	PSMT condition	Information about sources of stress related chemotherapy	Training in breathing techniques (with therapist in PSMT or video/audio in SSMT) Guided practice of progressive muscle relaxation, combined with guided imagery Combined with muscle relaxation, presented via script/audio Identification and rehearsal of coping self‐talk phrases	Using abdominal breathing before and after chemotherapy Practicing relaxation exercices as instructed Using imagery to manage stress Saying coping self‐statements during distressing situations	Prescribed daily practice of the three techniques before the start of chemotherapy and audio recorded to practice daily before chemotherapy and short meeting before the first chemotherapy cycle to answer questions and encourage the use of techniques learned.
	SSMT condition	Short information session before the chemotherapy, packet (e.g., prerecorded videotape and 12‐page booklet entitled “Coping With Chemotherapy”)			Recommendations in the videotape and booklet for practicing the techniques before the start of chemotherapy and using them the start of the treatment. Intersperd were provided by former chemotherapy patients about the benefits for mental and physical well‐being by using the stress management techniques. Short meeting before the first chemotherapy cycle to answer questions and encourage the use of techniques learned.
Nápoles et al. (2015)	Non‐specific psychosocial intervention	Managing the emotional impact of cancer (week 1) Finding the cancer information you need (week 2) Getting the support you need (week 3)	Psychoeducation on common reactions to a cancer diagnosis, sign of depression, tracking symptoms, deep breathing, breast cancer and breast cancer diagnostic test (week 1) Peer‐led coaching and informational support (week 2) Discussion of social support and communication techniques (week 3) Modeling and practicing cognitive reframing (week 4) Discussion and exercises on mood regulation (week 5) Demonstration of relaxation and pacing techniques (week 6) Activity planning and behavioral activation (week 7)	Recognizing emotional and physical reactions to diagnosis, emotional self‐monitoring. Tracking symptoms; deep breathing (week 1) Communicating needs to clinicians, participating in medical decision. Effective communication, asking for a medical interpreter (week 2). Talking about cancer with others; asking for help; faith and prayer (week 3) Identifying helpful and unhelpful thoughts (week 4) Managing thoughts and mood with reframing and self‐talk (week 5) Stress management. Using relaxation and pacing techniques (week 6) Managing activities that affect mood (week 7)	Setting goals—Part I (e.g., Managing activities, increasing pleasant activities, use of laughter, distraction techniques, goal‐setting) (week 7)—Setting goals (setting self‐care goals for the future; closing ceremony) (week 8)
O'Toole et al. (2020)	ERT‐C	Psychoeducation on metacognition followed by cognitive reappraisal training, decentring via reflection and mindfulness (session 1–4)	Psychoeducation and emotional support (session 1–4) Mindfulness training (4 practices); experiential exercises in attentional focus (Sessions 1–4) Exposure exercises targeting motivational conflicts (Sessions 5–8)	Attention regulation (shifting/sustaining attention on difficult experiences) (Session 1–4) Applying regulation in emotionally challenging contexts (Session 5–8)	Pursuing personally meaningful goals despite discomfort and choosing action aligned with values despite worry or avoidance
Smith et al. (2023)	CM	Psychoeducation on emotional processes Understanding the function of emotions—(e.g., adaptive functions of emotions, harmful beliefs about emotions, exploring the three components: cognitions, physical sensations/emotions, behaviors) (module 1 Understanding emotion)	Psychoeducation on emotional processes; emotion tracking exercises (module 1 “Understanding emotion”) Guided mindfulness practices and present‐focused attention techniques (module 2 Mindful Emotion Awareness) Cognitive restructuring, thought challenging, and planning exercises (module 3 Flexible Thinking) Behavioral activation, exposure to avoided contexts, and values clarification (module 4‐ Doing Things Differently)	Enhancing present‐moment awareness of emotions (module 2 Mindful Emotion Awareness) Developing cognitive flexibility (e.g., identifying cognitive distortions and learning how to challenge them) (module 3 Flexible Thinking) Engaging and training to replace unhelpful emotion‐driven behaviors with healthier “alternative actions” (module 4‐Doing Things Differently)	Guided mindfulness practices (i.e., learning mindfulness and non‐judgmental acceptance of emotions by practicing daily grounding techniques and 10‐min guided audio sessions)
Thilges et al. (2023)	CBI	Psychoeducation (e, g. depression, anxiety, common states mood) Discussion of patient information and emotional need (session 1—Introduction and Psychoeducation)	Psychoeducation on mood symptoms and emotional normalization (session 1—Introduction and Psychoeducation) Cognitive restructuring (e.g., reframing, thought stopping) (session 2—Cognitive Restructuring) Diaphragmatic breathing, progressive muscle relaxation, guided imagery (session 3—Relaxation Techniques) Activity scheduling using behavioral activation worksheets (session 4—Behavioral Activation) Assertiveness training and behavioral rehearsal (session 5—Communication skills) Behavioral education on health behaviors (session 6—Self‐care and health behaviors)	Recognizing emotional states (depression, anxiety, distress) (session 1 Introduction and Psychoeducation) Challenging and modifying distressing thoughts (session 2—Cognitive Restructuring) Managing physiological stress and arousal (session 3—Relaxation Techniques) Increasing engagement in positive activities (session 4—Behavioral Activation) Communicating emotional and informational needs (session 5—Communication skills) Maintaining self‐care (session 6—Self care and Health behaviors)	Review of prior techniques and relapse prevention planning (e.g., consolidating gains and preparing for future challenges and anticipating future gaps and sustaining progress) (session 7 Relapse prevention)
Farnoodimehr et al. (2021)	UP‐CBT	Increasing the motivation and describing the intervention (session 1) Psychoeducation on Emotions (session 2–3)	Presentation of treatment, increasing commitment (session 1) Education on emotions, their function and impact (Session 2–3—Psychoeducation on Emotions) Training in mindful awareness of current emotional experiences (Session 4) Cognitive reappraisal exercises, increasing flexible thinking (session 5) avoiding emotions and emotion‐driven behaviors (session 6) Exercises to face and tolerate bodily sensations linked to emotions (session 7) Interoceptive and situational exposure techniques (sessions 8–11—Emotional exposure)	Motivation, engagement (session 1) Recognizing emotional experiences (session 2–3) Mindful attention to emotion (session 4) Reappraisal, psychological flexibility (session 5) Awareness of avoidance patterns (session 6) Interoceptive awareness (session 7) Emotional processing (sessions 8–11)	Relapse prevention (session 12)

Abbreviations: CBI, Cognitive Behavioral Intervention; CM, CanCopeMind; EFT‐CA, Emotion Focused Therapy‐Caregiver; ERT‐C, Emotional Regulation Training‐Caregivers; NS, Not Specified; PSMT, Professionally administered stress management training; SSMT, Self‐administered stress management training; UP‐CBT, Unified Protocol‐Cognitive Behavioral Therapy.

Despite structural variations, most interventions followed a similar three‐phase model: (1) Information dissemination, (2) development of adaptive capacities (i.e., skills and strategies) via specific therapeutic processes, and (3) reinforcement of these capacities. While not always explicitly stated, this structure was consistently observed.

#### 3.6.1 Information Dissemination

All interventions included an information phase except one [35]. This phase addressed a range of topics, such as understanding emotions [39, 40, 41], the emotional impact of cancer [36] or basic metacognition concepts (i.e., decentering from and reappraisal of emotional experiences) [42]. One study [39] emphasized the importance of clearly stating intervention goals to enhance participant motivation. Clarifying these goals early in the intervention could help give meaning to the program and increase participant motivation.

#### 3.6.2 Adaptive Capacities

All interventions targeted the development of adaptive capacities, understood as both skills (i.e., psychological abilities developed by participants) and strategies (i.e., behavioral or cognitive actions applied in daily life). The interventions sought to support these capacities using specific therapeutic processes (i.e., the techniques or content delivered within sessions). Given the often‐blurred line between skills and strategies in the reviewed interventions, we grouped them under the broader category of “adaptive capacities” (see Table 4), which includes both the capacities trained and the concrete actions promoted. The intervention content was designed to influence ER mechanisms.

#### 3.6.3 Emotion Regulation Mechanisms

The targeted adaptative capacities included emotional capacities (e.g., identifying emotions, emotional self‐monitoring, stress management, present‐moment awareness of emotion, recognizing emotional states [23, 35, 37, 39, 41]), cognitive capacities (e.g., attentional regulation, identifying helpful and unhelpful thoughts, cognitive flexibility [23, 35, 37, 39, 40, 41, 42]) and behavioral and social capacities (e.g., behavioral activation aligned with personal values, communicating needs to clinicians, seeking support for engagement in positive activities, maintaining self‐care, expressing emotional and informational needs, motivation [23, 37, 39, 40, 41]). Interventions promoted adaptive capacities by incorporating ER exercises such as visualization [16] breathing and relaxation techniques [37, 38, 40, 41], mindfulness [42] and stress management strategies such as reducing avoidance [38, 40]. Some strategies reinforced internal adaptive skills, such as recognizing emotional and physical reactions and communicating effectively with healthcare providers to better manage cancer [37].

#### 3.6.4 Consolidation

Finally, several studies included between‐session tasks to consolidate the adaptive capacities developed during the intervention. These tasks included listening to audio recordings [38, 40], practicing exercises introduced during the sessions [40], or pursuing personally meaningful goals [42]. Two studies [39, 41] implemented a dedicated relapse prevention phase at the end of the intervention, aiming to consolidate progress and anticipate future challenges. While not explicitly labeled “relapse prevention,” the final session in Nápoles et al.'s interventions [37] included elements consistent with this aim, such as reviewing key content and setting long‐term self‐care goals, suggesting an intention to promote the consolidation and sustainability of acquired adaptive capacities.

The development of these adaptive capacities supports ER mechanisms, which, in turn, positively impact psychological processes and psychosocial outcomes.

### 3.7 Intervention Efficacy

Table 5 summarizes the efficacy of interventions in improving ER, related psychological processes (e.g., psychological flexibility, decentering, reappraisal, mindfulness, avoidance, and other regulation strategies), and psychosocial outcomes (i.e., distress, anxiety, depression, fear of cancer recurrence, quality of life, emotional well‐being, or relatives' burden). Three studies assessed ER as a primary outcome [23, 39, 42], while others focused on related processes like mindfulness or avoidance [23, 37, 39, 40]. Several also evaluated broader psychological outcomes such as anxiety, depression [37, 39, 41], fear of cancer recurrence [35], quality of life [37], emotional well‐being [42] and caregiver burden [23, 42].

**TABLE 5:** Intervention effects on emotion regulation and associated psychological outcomes.

Authors	Measure	Pre (T1) mean (SD/95% CI)	Mid (T2)	Post (T3) mean (SD/95% CI)	Group (*F*)/mean (SD)	Change	*p*	Effect size
Almeida et al. (2022)	CORE‐OM	M = 18.38 (5.15)	NS	M = 13.56 (5.76)	NS	NS	< 0.001	*d* = 0.88
	PQ	M = 5.78 (0.52)	NS	M = 4.89 (1.56)	NS	NS	< 0.001	*d* = 1.53
	PQ‐FCR	M = 6.15 (0.95)	NS	M = 4.89 (1.56)	NS	NS	0.001	*d* = 0.98
Applebaum et al. (2020)	DERS	81.94 (74.32, 89.55)	NS	68.37 (61.17, 75.57)	NS	−13.42	0.001	*g* = 0.68
	FFMQ	126.87 (121.0, 132.7)	NS	143.95 (134.7, 153.2)	NS	+17.42	< 0.001	*g* = −0.92
	HADS total	18.87 (16.51, 21.24)	NS	14.74 (12.28, 17.19)	NS	−4.58	0.009	*g* = 0.65
	HADS‐a	11.55 (10.36, 12.74)	NS	9.37 (7.98, 10.76)	NS	−2.74	0.005	*g* = 0.66
	HADS‐d	7.32 (5.79, 8.85)	NS	5.37 (4.02, 6.72)	NS	−1.84	0.046	*g* = 0.49
	CRA	79.65 (76.4, 82.89)	NS	78.35 (75.08, 81.62)	NS	1.45	0.531	*g* = 0.15
	RRS	45.74 (41.65, 49.83)	NS	42.68 (39.58, 45.79)	NS	−3.89	0.006	*g* = 0.36
	PSWQ	54.77 (49.95, 59.6)	NS	48.68 (43.88, 53.49)	NS	−8.79	< 0.001	*g* = 0.47
Jacobsen et al.(2002)^a^	STAI‐S	NS	NS	NS	*F* = 0.11^b^/*F* = 5.18^c^	NS	0.74/0.02	NS
	CES‐D	NS	NS	NS	*F* = 2.51^b^/*F* = 7.01^c^		0.11/0.009	NS
	SF‐36 (Role emotional)	NS	NS	NS	*F* = 0.03^b^/*F* = 0.37^c^		0.87/0.54	
Nápoles et al. (2015)^d^	FACT‐B	66.46 (16.92)	77.24 (15.13)	80.64 (13.64)	M = 68.83 (15.33) (t1)	NS	0.37 (t1)	NS
					M = 74.39 (15.34) (t2)		0.37 (t2)	NS
					M = 77.02 (15.62) (t3)		0.174 (t3)	NS
	BSI‐d	0.93 (0.84)	0.46 (0.59)	0.38 (0.48)	M = 0.75 (0.76) (t1)	NS	0.164 (t1)	NS
					M = 0.52 (0.63) (t2)		0.531 (t2)	NS
					M = 0.46 (0.62) (t3)		0.355 (t3)	NS
	BSI‐a	0.93 (0.84)	0.48 (0.66)	0.39 (0.53)	M = 1.01 (0.88) (t1)	NS	0.577 (t1)	NS
					M = 0.60 (0.73) (t2)		0.32 (t2)	NS
					M = 0.58 (0.76) (t3)		0.465 (t3)	NS
O'Toole et al. (2020)^e^	DERS	86.9 (20.7)	80.7 (22.5)	72.1 (20.8)	M = 92.8 (24.2) (t1)		0.002	*g* = 0.77
					M = 89.9 (24.2) (t2)			
	ERQ‐R	26.4 (5.7)	27.1 (5.4)	29.0 (6.1)	M = 27.1 (6.9) (t1)		0.031	*g* = 0.43
					M = 26.2 (6.1) (t2)			
	EQ	33.9 (5.8)	35.6 (6.1)	39.1 (6.2)	M = 34.3 (9.4) (t1)		< 0.001	*g* = 0.93
					M = 34.2 (8.0) (t2)			
	FFMQ	125.5 (15.2)	131.0 (16.9)	139.0 (18.2)	M = 118.9 (16.1) (t1)		< 0.001	*g* = 0.92
					M = 120.0 (19.4) (t2)			
	HADS total^f^	20.5 (7.1)	15.4 (6.8)	13.1 (7.3)	M = 19.2 (6.6) (t1)		< 0.001	*g* = 0.86
					M = 17.4 (6.6) (t2)			
	HADS total^g^	12.1 (6.3)	NS	8.9 (5.0)	M = 13.0 (13.8) (t1)		0.672	*g* = 0.14
					M = 11.6 (8.4) (t2)			
	(WHO)‐5^h^	9.2 (5.3)	12.5 (5.0)	15.5 (4.7)	M = 10.2 (5.4) (t1)		0.001	*g* = 0.79
					M = 12.3 (5.6) (t2)			
	EORTC QLQ‐C30^i^	53.4 (17)	NS	67.2 (18.6)	M = 57.2 (21.1) (t1)		0.019	*g* = 0.88
					M = 54.5 (26.7) (t2)			
	CRA	35.7 (10.4)	31.6 (11.0)	29.9 (10.5)	M = 35.0 (11.4) (t1)		0.006	*g* = 0.55
					M = 34.3 (11.3) (t2)			
	PSWQ	50.9 (10.2)	49.1 (9.4)	40.8 (8.1)	M = 52.6 (10.0) (t1)		< 0.001	*g* = 0.96
					M = 51.4 (8.8) (t2)			
	RRS‐B	10.8 (3.2)	9.0 (3.1)	8.7 (2.8)	M = 11.2 (3.4) (t1)		0.220	*g* = 0.24
					M = 10.0 (3.5) (t2)			
Smith et al. (2023)^j^	BES	−5.35 (−9.56, −1.12)^k^	−6.12 (−11.28, −0.90)^l^	3.58 (0.57, 6.58)^m^	NS		0.005/0.009/0.008	SMD = 0.34/0.31/0.25
	SMQ	7.15 (2.56, 11.75)^k^	8.82 (3.91, 13.74)^l^	6.07 (2.32, 9.82)^m^	NS		< 0.001	SMD = 0.47/0.54/0.38
	UP‐CSQ	1.63 (0.01, 3.38)^k^	1.85 (0.14, 3.59)^l^	1.07 (−0.03, 2.23)^m^	NS		0.036/0.016/0.039	SMD = 0.31/0.38/0.22
	CERQ‐C	−0.69 (−1.47, 0.02)^k^	−71 (−1.67, 0.25)^l^	−0.06 (−0.36, 0.29)^m^	NS		0.049/0.109/0.368	SMD = 0.29/0.26/0.02
	CERQ‐R	0.69 (−0.51, 1.89)^k^	0.50 (−73, 1.73)^l^	0.08 (−0.87, 1.03)^m^	NS		0.195/0.358/0.844	SMD = 0.19/0.15/0.02
	MEAQ‐30	−8.58 (−14.05, −3.05)^k^	−13.89 (−20.59, −7.19)^l^	−1.56 (−5.30, 2.21)^m^	NS		0.001/0.001/0.344	SMD = 0.42/0.69/0.08
Thilges et al. (2023)^n^	BSI‐d	49.44	44.68	43.57	UC depression M = 50.85 (t1) M = 49.46 (t2) M = 52.23 (t3)	NS	NS	NS
	BSI‐a	44.20	43.49	42.11	UC Anxiety M = 44.20 (t1) M = 43.56 (t2) M = 47.98 (t3)	NS	NS	NS
	BDI‐II	6.85	5.54	7.02	UC depression M = 8.24 (t1) M = 6.58 (t2) M = 8.40 (t3)	NS	NS	NS
	FACT‐H&N—Emotional well being	19.40	20.35	20.60	UC—Emotional well being M = 19.93 (t1) M = 19.80 (t2) M = 19.71 (t3)	NS	NS	NS
Farnoodimehr^o^ et al. (2021)	DASS‐d	13.05 (1.73)	7.49 (1.51)	8.87 (1.74)	NS	NS	< 0.01 (*F* = 49.36)	NS
	DASS‐a	14.22 (1.47)	10.72 (1.10)	11.27 (1.36)	NS	NS	< 0.01 (*F* = 312.80)	NS
	DASS‐s	19.61 (2.19)	15.11 (1.78)	15.83 (1.79)	NS	NS	< 0.01 (*F* = 72.33)	NS
	ERQ‐ r	19.11 (1.27)	24.38 (1.91)	24.05 (1.64)	NS	NS	< 0.01 (*F* = 47.54)	NS
	ERQ‐s	21.38 (1.24)	16.83 (1.46)	17.38 (1.41)	NS	NS	< 0.01 (*F* = 85.93)	NS
	AAQ‐II	30.05 (1.76)	22.50 (1.68)	22.11 (1.64)	NS	NS	< 0.01 (*F* = 115.47)	NS

Abbreviations: AAQ‐II, Acceptance and Action Questionnaire—II; BDI‐II, Beck Depression Inventory; BSI, Brief Symptom Inventory; CERQ, Cognitive Emotion Regulation Questionnaire; CES‐D, Center for Epidemiologic Studies Depression Scale; CORE‐OM, Clinical Outcomes in Routine Evaluation—Outcome Measure; CRA, Caregiver Reaction Assessment; DASS, Depression Anxiety Stress Scales; DERS, Difficulties in Emotion Regulation Scale; EORTC QLQ‐C30, European Organization for Research and Treatment of Cancer Quality of Life Questionnaire—Core 30; EQ, Experiences Questionnaire; ERQ, Emotion Regulation Questionnaire; FACT‐B, Functional Assessment of Cancer Therapy—Breast; FACT‐HNS, Functional Assessment of Cancer Therapy—Head & Neck Scale; FFMQ, Five Facet Mindfulness Questionnaire; HADS, Hospital Anxiety and Depression Scale; MEAQ‐30, Multidimensional Experiential Avoidance Questionnaire—30 items; NS, Not specified; PQ, Personal Questionnaire; PQ‐FCR, Personal Questionnaire—Fear of Cancer Recurrence; PSWQ, Penn State Worry Questionnaire; RRS‐B, Ruminative Response Style‐Brooding subscale; SF‐36, Study 26‐Item Short Form; SMQ, Stress Management Questionnaire; STAI‐S, State–Trait Anxiety Inventory—State form; UP‐CSQ, Unified Protocol—Coping Skills Questionnaire; WHO‐5, World Health Organization‐5 Well Being Index.

^a^ The *F*‐values (*F* = 6.26 for CES‐D and *F* = 4.09 for STAI‐S) represent the interaction effect of group × time across all three groups (SSMT, PSMT, UCO). Within‐group *t*‐tests indicate significant reductions in depression and anxiety in the SSMT group, and a modest improvement in anxiety in the PSMT group.

^b^ UCO with PSMT.

^c^ UCO with SSMT.

^d^ The *p* value was calculated for intergroup comparison. Measurements were carried out at 3 and 6 months.

^e^ The *p* value and *g* were calculated for intergroup comparison. Measurements were carried out at baseline, 4 and 8 weeks.

^f^ Caregivers outcomes.

^g^ Patients outcomes.

^h^ Caregivers outcomes.

^i^ Patients outcomes.

^j^ The results concern only the CanCope Mind intervention, which targets emotion regulation.

^k^ Baseline to post‐intervention, T0–T4.

^l^ Baseline to follow‐up, T0–T5.

^m^ Pre‐to‐post modules on target outcomes.

^n^ Measurement were carried out at 3, 6 and 12 months.

^o^ Timing of assessments (e.g., post‐test ≈ 3 months, follow‐up ≈ 4 months) inferred from the intervention schedule and the reported 4‐week follow‐up interval. The *p* value was calculated for intergroup comparison.

Overall, the interventions showed significant improvements in ER and psychological outcomes. However, ERT‐C did not significantly reduce caregiver burden [22], and the Nuevo Amanecer interventions [37] a culturally tailored cognitive‐behavioral stress management program delivered by trained Latina breast cancer survivors, likewise reported no significant effects on quality of life, depression, or anxiety. The three studies explicitly assessing ER [23, 39, 42] all reported significant post‐intervention improvements, suggesting that targeting these processes can lead to clinically meaningful emotional changes. Other interventions, such as mindfulness, cognitive reappraisal, or reduced experiential avoidance [23, 39, 42] led to improvements in psychological processes related to ER. These process changes are important mechanisms that can contribute to better emotional adjustment. Additionally, several studies showed beneficial effects on mental health indicators, including reductions in anxiety and depressive symptoms [37, 39, 41] decreased fear of cancer recurrence [35], and improved quality of life [37] and emotional well‐being [41].

Notably, although O'Toole et al.'s caregiver‐focused intervention [42] improved cancer patients' quality of life, despite no direct impact on their distress, suggesting that enhancing relatives' ER may indirectly benefit patients.

**FIGURE 2:**
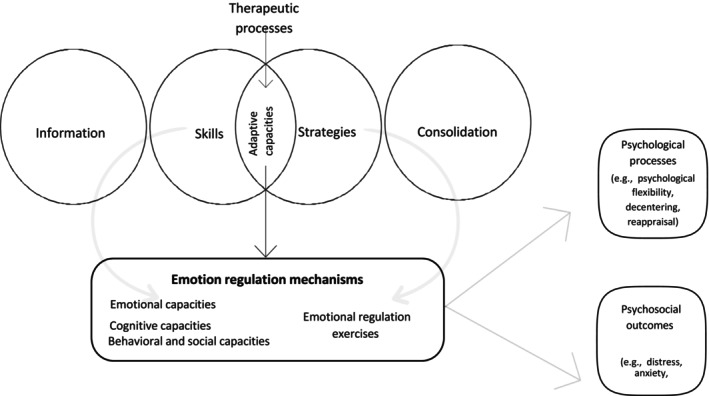
Pathways from intervention components to emotion regulation mechanisms and their impact on psychological processes and psychosocial outcomes.

In Figure 2, we provide an overview of the pathways linking intervention components, ER mechanisms, and clinical efficacy in psychological processes and psychosocial outcomes.

### 3.8 Intervention Acceptability

Only two studies reported qualitative data on the acceptability of the interventions following completion. They used semi‐structured interviews [23] or the Helpful Aspects of Therapy questionnaire [35] to gather qualitative feedback from participants on what they found helpful during the sessions.

Participants found the individual sessions helpful for processing emotions but suggested including at least one dyadic session with patients and relatives to practice skills together [23]. They also highlighted the challenge of applying concepts like courage and compassion to daily life and preferred in‐person weekly one‐hour sessions [23]. Participants also valued EFT‐Ca for helping express and transform fear of cancer recurrence, reporting reduced anxiety, increased safety, and a renewed hope and self‐efficacy [35].

### 3.9 Determinants of Attrition and Intervention Efficacy

Attrition rates varied widely, from 7.5% [39] to 74.47% [41], showing challenges for long‐term follow‐up. One study did not report attrition rates [35]. Nápoles et al.'s interventions [37] reported excellent retention, 93% in the intervention group and 97% in controls, with 82% completing most sessions. This notably high retention rate may be explained by several contextual and methodological factors. High retention may be due to delivering the intervention in a community setting rather than hospitals, use of trained peer Latina breast cancer survivors instead of clinicians, and logistical flexibility like make‐up sessions. These factors emphasize cultural tailoring, peer involvement, and accessibility as key for retention and acceptability.

Attrition was also linked to participant factors: lower initial quality of life [38] and higher stress [40] predicted dropout, though O'Toole et al. found the opposite with stress. Older age [42] and male gender [38] were associated with higher attrition, but some studies found no gender effect of higher dropout among younger participants [40, 41]. Cancer stage showed no impact [41]. Common reasons for attrition included lack of time or feeling overwhelmed [42], altered health status [35], and burden on relatives [23].

### 3.10 GRADE Assessment—Certainty of Evidence

Complete results for the GRADE assessment are presented in Appendix C. For the indirectness criterion, we noted very low concern across all interventions, with all included studies answering our PICO question appropriately. Inconsistency was marked “not applicable” (NA) for all outcomes because this criterion assessed variability in results across multiple studies for the same outcome, an evaluation that was not feasible here since most outcomes were informed by a single study or by studies too heterogeneous to be compared meaningfully. Similarly, publication bias could not be assessed (marked NA) due to the limited number of studies per outcome.

The independent assessment conducted by AN and checked by KL resulted in a distribution of recommendations across varying levels of evidence quality. In total, four outcomes were supported by high‐quality evidence, including three critical outcomes from interventions designed for relatives or caregivers and one important outcome; 20 outcomes were supported by moderate‐quality evidence (critical: *n* = 9; important: *n* = 11); 18 outcomes were supported by low‐quality evidence (critical: *n* = 2; important: *n* = 16); and one outcome (i.e., caregiver burden) was supported by very low‐quality evidence.

High‐quality evidence supported the use of an ERT‐C (*n* = 3) or Unified Protocol for Cognitive Behavioral Therapy (UP‐CBT, *n* = 1) intervention for various outcomes: emotion regulation difficulties, mindfulness, decentering, and depression.

## 4 Discussion

This systematic review identified individual interventions targeting ER in patients with cancer and their relatives and described their modalities and efficacy. Despite structural variations, the interventions shared common components: an initial information phase, therapeutic processes to develop adaptive capacities (i.e., skills and strategies), and a reinforcement phase involving between‐session tasks or relapse prevention. Most showed significant effects for ER, psychological outcomes, emotional well‐being, or quality of life among both individuals affected by cancer and their relatives.

However, high attrition rates highlight the need for future research to develop and refine methodological strategies that enhance participant retention. Additionally, acceptability data remain scarce, with only two studies reporting qualitative data. Future research should systematically assess acceptability to enhance participant engagement.

### 4.1 Description of Interventions

Most of the included studies used CBT techniques (i.e., relaxation, guided imagery, systematic desensitization, breathing exercises, biofeedback, and cognitive restructuring), which is recommended in psycho‐oncology for its brevity and focus on current illness‐related issues [43]. Evidence suggested that CBT integrates ER as a component of a broader treatment, making it difficult to isolate the specific part of the intervention that influences ER or contributes to overall clinical or functional improvement [44]. Interventions commonly combined information dissemination with developing emotional, cognitive, behavioral, and social capacities through in‐session and between‐session activities. Studies have shown that combining informational and emotional interventions is a prerequisite for adapting to an illness [45, 46], influencing emotional adjustment and psychological outcomes. Others have suggested that an effective intervention design aimed at supporting patients with cancer should be based on a three‐component theory: information provision, behavioral therapies, and emotional support [47, 48], though some suggest that solely informational or CBT‐based approaches may suffice [49, 50].

While our review focused on individual ER interventions, group interventions also exist and appear equally effective [43, 51, 52]. The group setting offers a unique therapeutic environment in which participants benefit from social support and shared experiences [53]. A study included in our review opted for an individual format (i.e., remote or in‐person), citing greater logistical flexibility and the potential to reduce participant attrition during intensive treatment [41]. Future research should explore how intervention format, setting, and population characteristics interact to influence outcomes.

### 4.2 Mechanistic Pathways and Intervention Efficacy

Three studies found no significant effects regarding quality of life, depression, and anxiety [37]; caregiver burden [23]; or patient distress, when the intervention was delivered indirectly via relatives [42]. Discrepancies can arise between the intervention target (outcome of interest) and the variables assessed (outcome assessed), which could explain the lack of significant results. For example, Applebaum et al.'s intervention [23] targeted ER, but assessed caregiver burden, revealing a misalignment between the intervention's focus and the outcome measured. Future research should ensure that intervention goals are clearly matched with outcome measures that are both relevant and sensitive.

Additionally, methodological issues also arose. Smith et al.'s CanCope Lifestyle control intervention [40] targeted diet and sleep, not ER, yet psychological outcomes were used for both CanCope arms, complicating attribution of effects. Similarly, Thilges et al. emphasized self‐care behaviors (e.g., oral hygiene, nutritional goals, and reducing tobacco and alcohol use) and quality of life rather than emotional outcomes [41]. These cases highlight the need for well‐defined mechanisms and outcome measures that accurately reflect intervention goals. Precise conceptualization of ER (i.e., its competencies and strategies) is essential to test mechanisms of change [44] and designing specific, effective targeted interventions. None of the reviewed studies conducted predictive analyses connecting ER variables (e.g., psychological flexibility, reappraisal, mindfulness) to outcomes like anxiety or depression. Nevertheless, evidence supports that strengthening adaptive ER skills prevents or reduces distress [54], since ER is a transdiagnostic factor in many disorders [44]. Future trials should explicitly test these associations and mechanisms. To address this point, studies have considered potential mediators or moderators of the efficacy of interventions. For example, Traeger et al. examined mediators like disease perception and perceived control, and moderators such as initial stress [55] and Boesen et al. found better effects in married patients [56]. Finally, interventions may indirectly benefit patient quality of life when relatives participate, with positive effects observed for both [57].

### 4.3 Attrition in the Studies

Attrition rates were high across studies, with contradictory explanations for dropouts. While Smith et al. observed that highly stressed patients drop out more often [40], O'Toole et al. found the opposite [42]. This discrepancy may relate to motivations for ER: some patients aim to reduce negative effects, while others use emotions adaptively. As Tamir argues, ER is not only driven by hedonic goals (reducing negative emotions) but can also serve instrumental or eudemonic purposes (e.g., preserving meaning or relationships) [58]. Despite these elements potentially explaining a limitation in the literature, they are experimentally based and require further studies in clinical settings.

Illness beliefs may also influence attrition. Cameron et al. showed that patients who believe their emotional distress affects the progression of their cancer are more likely to participate [59]. In such cases, the motivation to regulate their emotions may be the fear of disease progression. Others suggested that causal attributions may be key to understanding ER motivations [60]. In parallel with motivations, the conceptualization of emotions should also be considered. Interventions reviewed mainly aimed to reduce negative emotions, but some studies suggest negative emotions can be adaptive. The concept of “mixed emotions” posits that positive and negative feelings can coexist simultaneously [61, 62].

Other determinants of attrition include resistance to change [63] highlighting the need to tailor interventions to individual needs, such as session number or offering individual support. Beyond conceptual factors influencing motivation, maintaining patient engagement remains challenging. A comprehensive understanding of attrition factors is crucial to develop effective retention strategies. None of the reviewed studies reported involving patients or caregivers in intervention design. Future research should consider individual characteristics, emotional trajectories, and timing within the care pathway [64], to reduce attrition. Offering interventions to patients with very high or very low anxiety may also affect outcomes; while evidence suggested patients with lower stress show greater emotional gains [55], others argue that baseline distress is necessary to observe significant effects [65]. Recommendations to address attrition include improving post‐intervention communication and adding booster sessions [51].

### 4.4 Limitations and Perspectives

One limitation of this review is the lack of clear ER definitions in many studies and insufficient distinction between skills and specific strategies. To address this conceptual ambiguity, we used the term “adaptive capacities” to cover both skills and strategies since the original studies often failed to distinguish between these concepts. Additionally, our synthesis methods were limited by the heterogeneity in and variable reporting of outcomes across studies, which prevented us from conducting a meta‐analysis and diminished the strength of conclusions drawn from the data. More broadly, the generalizability of our findings was limited by the exclusion of dyadic and group‐based interventions. Although our focus on individual interventions allowed for a more consistent synthesis of therapeutic processes, this choice may have resulted in our exclusion of valuable insights into relational and systemic aspects of ER. Future studies should expand their scope to include such formats and establish a broad spectrum of possible interventions. Integrating ER models [9, 66, 67] could strengthen intervention design. Moreover, several studies lacked transparency regarding intervention content; for instance, O'Toole et al. did not specify the psychoeducational components [42]. Further research is needed to clarify how therapeutic processes target ER skills and strategies, termed adaptive capacities here, due to unclear distinctions across interventions. Future studies should clearly separate skills (internal capacities) from strategies (their application) to better understand change mechanisms. None reported co‐creation with participants, possibly explaining limited transferability and high attrition. In this vein, a recent feasibility study provided a standardized manual specifically designed to meet the needs of relatives [68]. The only peer‐led intervention [37] identified in our review, facilitated by breast cancer survivors, demonstrated a substantially lower rate of participant dropout. Future research should co‐develop interventions with stakeholders to enhance relevance and retention. Lastly, only two studies addressed relatives, and few assessed ecological validity or acceptability.

### 4.5 Recommendation for Clinical Practice

Based on our GRADE assessment, some interventions, namely the ERT‐C for caregivers [42] and UP‐CBT [39] for patients, show promise for implementation in clinical settings, with the understanding that evidence quality varies between studies and additional replication is needed. Clinicians should interpret these recommendations within the context of available resources, patient preferences, and local expertise while referring to ongoing research that may refine these recommendations.

Future research should prioritize larger, better‐powered RCTs with the more consistent reporting of standardized effect sizes and confidence intervals to improve precision and comparability across studies. Follow‐up assessments are also needed to evaluate long‐term effects, and the replication of promising interventions such as the ERT‐C and UP‐CBT among cancer patients and their relatives could increase confidence in their effectiveness.

## 5 Conclusions

This systematic review provides insights for the strategic development and evaluation of interventions targeting ER and psychological health outcomes in patients with cancer and their relatives. Given the high prevalence of psychological disorders in this population, targeting ER is crucial for both preventing and reducing the psychological impact, particularly when these psychological issues are already present at baseline. To develop and evaluate new interventions, future studies must further refine the concept of ER, clearly specify the competencies and strategies they target, thereby elucidating the mechanisms of change, and rigorously assess intervention efficacy. Finally, the high attrition rate across the reviewed studies highlights the need to strengthen participant engagement to maximize the impact of interventions.

## Author Contributions

Conceptualization: Ambre Naeyaert, Valentyn Fournier Laura Caton, Marie‐Charlotte Gandolphe, Christelle Duprez, Lisa Laroussi‐Libeault, Marie Vander Haegen, Pascal Antoine, Kristopher Lamore. Data curation: Ambre Naeyaert, Valentyn Fournier, Laura Caton, Marie‐Charlotte Gandolphe, Marie Vander Haegen, Kristopher Lamore. Formal analysis: Ambre Naeyaert, Valentyn Fournier, Laura Caton. Funding acquisition: Kristopher Lamore. Investigation: Ambre Naeyaert, Valentyn Fournier, Laura Caton, Kristopher Lamore. Methodology: Ambre Naeyaert, Valentyn Fournier, Laura Caton, Marie‐Charlotte Gandolphe, Marie Vander Haegen, Kristopher Lamore. Project administration: Ambre Naeyaert, Valentyn Fournier, Kristopher Lamore. Resources: Ambre Naeyaert, Valentyn Fournier, Laura Caton, Kristopher Lamore. Software: Ambre Naeyaert, Valentyn Fournier, Laura Caton, Kristopher Lamore. Supervision: Kristopher Lamore. Validation: Ambre Naeyaert, Valentyn Fournier, Laura Caton, Kristopher Lamore. Visualization: Ambre Naeyaert, Valentyn Fournier. Writing Original Draft: Ambre Naeyaert, Valentyn Fournier. Writing – Review and Editing: Ambre Naeyaert, Valentyn Fournier, Laura Caton, Marie‐Charlotte Gandolphe, Christelle Duprez, Lisa Laroussi‐Libeault, Marie Vander Haegen, Pascal Antoine, Kristopher Lamore.

## Ethics Statement

The authors have nothing to report.

## Conflicts of Interest

The authors declare no conflicts of interest.

## Supporting information


**Appendix A** Search strategy.

**Appendix B** Review.

**Appendix C** GRADE assessment of evidence certainty for critical and important outcomes.

## Data Availability

All data generated or analyzed during this study are included in this published article and its Supporting Information files. The review protocol was pre‐registered in PROSPERO (https://www.crd.york.ac.uk/PROSPERO/view/CRD42023484110).
