# Supplementary material for: Emotion Regulation Interventions for Cancer Patients and Their Relatives: A Systematic Review
Source: Cancer Med. 2026 Feb 12;15(2):e71514. doi: 10.1002/cam4.71514 (PMC12900081; doi:10.1002/cam4.71514)
Supplement: Supplementary file 1 — Appendix A Search strategy. [file CAM4-15-e71514-s001.docx]

(emotion* OR affect* OR mood* OR feel*)

AND

(regulat* OR dysregulat* OR intelligence OR manag* OR competence* OR skill* OR strateg* OR reapparaisal OR acceptance OR modification OR suppression OR avoidance OR identification OR reactivity OR awareness OR consciousness OR coping OR expression)

AND

(intervention* OR training OR program* OR trial* OR sessions)

AND

(tumour* OR tumor* OR cancer* OR oncolog* OR carcinoma* OR maligna* OR neoplasm* OR metasta* OR *sarcoma* OR leukaemia OR lymphoma OR mesothelioma OR myeloma OR *blastoma OR melanoma)

NOT

(pediatr* OR child* OR palliative OR end-of-life)

NOT

("systematic review" OR "meta-analysis" OR "meta-synthesis" OR "narrative review")

NOT

(“case reports” or “comment” or “editorial” or “lectures” or “legal cases” or “legislation” or “letter”)
