# Supplementary material for: Emotion Regulation Interventions for Cancer Patients and Their Relatives: A Systematic Review
Source: Cancer Med. 2026 Feb 12;15(2):e71514. doi: 10.1002/cam4.71514 (PMC12900081; doi:10.1002/cam4.71514)
Supplement: Supplementary file 3 — Appendix C GRADE assessment of evidence certainty for critical and important outcomes. [file CAM4-15-e71514-s003.docx]

Appendix C. GRADE Assessment of Evidence Certainty for Critical and Important Outcomes

| Outcome | Assessment | Importance | Population  (patients and/or relatives) | Intervention | Comparator | First author / N participants | Global result (Mean, SMD, g...) | IC95% / p-value | GRADE criteria (H/M/L/VL) | | | | | | Rate of the quality of each outcome (H/M/L/VL) | Comments |
| --- | --- | --- | --- | --- | --- | --- | --- | --- | --- | --- | --- | --- | --- | --- | --- | --- |
|  |  |  |  |  |  |  |  |  | Risk of bias | Imprecision | Inconsistency | Indirectness | Effect size | Publication bias |  |  |
| Emotion regulation difficulties | DERS | Critical | Relatives | ERT-C | Pre-post intervention | Applebaum/ N=22 | Pre : M=81.94, Post : M=68.37, g=0.68 | Pre : (CI 95% 74.32,89.55),Post : (CI 95% 61.17,75.57) / .001 | Moderate | Moderate concern | NA | Very low concern | Moderate | NA | Moderate | The effect estimate is based on a relatively small sample size, and confidence intervals were not reported, which limits the precision of the findings.While available studies assessing this outcome generally show positive effects, inconsistency cannot be adequately assessed here because of heterogeneity in study designs (e.g., RCT vs. pre-post).The outcome shows a moderate-to-large effect size (g = 0.79, p = .001), suggesting a clinically meaningful impact of the intervention compared to the control group.The certainty of evidence is downgraded due to the small sample size and the lack of confidence intervals, although the observed effect is promising. Publication bias, could not be assessed because of variability in designs and the limited number of studies available for this outcome. |
| Emotion regulation difficulties | DERS | Critical | Relatives | ERT-C | Waitlist | O'Toole / N=43/37 (ERT-C/Waitlist) | Intervention - Pre : M=86.9 Post: M=72.1 / Control group - Pre : M= 92.8, Post: M=89.9 g=0.77 | NR / .002 | Very low concern | Moderate concern | NA | Very low concern | High | NA | High | The RCT design strengthens the overall quality of evidence compared to pre–post designs. The relatively small sample size and the lack of reported confidence intervals reduce the precision of the estimate, although the observed effect was statistically significant. Cannot be adequately assessed due to the heterogeneity of study designs across the available literature.The intervention showed a moderate-to-large effect on the outcome, suggesting a potentially meaningful clinical benefit. Publication bias, could not be assessed because of the limited number of studies and methodological variability across the evidence base. |
| Mindfulness | FFMQ | Critical | Relatives | ERT-C | Pre-post intervention | Applebaum / N=22 | Pre : M=126.87, Post : M=143.95, g= 0.92 | Pre : (CI 95% 121.0,132.7), Post : (CI95% 134.7,153.2) / p < .001 | Moderate | Moderate concern | NA | Very low concern | High | NA | Moderate | The pre–post design lowers the overall quality of evidence despite the presence of a notable observed effect.The small sample size and the relatively wide confidence interval contribute to moderate imprecision in the effect estimate.Although studies assessing mindfulness report significant improvements, inconsistency could not be evaluated due to differences in study designs across the available evidence.Publication bias, could not be assessed because of the heterogeneity and limited number of studies. |
| Mindfulness | FFMQ | Critical | Relatives | ERT-C | Waitlist | O'Toole /N=43/37 (ERT-C/Waitlist) | Intervention - Pre : M=125.5, Post : M=139.0, Control group - Pre : M= 118.9, Post : 120.0, g=0.92 | NR / <.001 | Very low concern | Moderate concern | NA | Very low concern | High | NA | High | The RCT design of the intervention strengthens the quality of evidence. However, the absence of reported confidence intervals warrants downgrading for imprecision. Inconsistency, could not be assessed due to the different study designs used to evaluate this outcome across the available evidence. Although the study reports a clear beneficial effect on mindfulness compared with the control group, the lack of precision limits our overall confidence in the estimate. Publication bias, could not be evaluated because of the heterogeneity and limited number of studies. |
| Cognitive reappraisal | ERQ-R | Critical | Relatives | ERT-C | Waitlist | O'Toole/ N=43/37 (ERT-C/Waitlist) | Intervention - Pre : M=26.4, Post : M=29.0, Control group - Pre : M=27.1, Post: M=26.2, g=0.43 | NR/.031 | Very low concern | Moderate concern | NA | Very low concern | Moderate | NA | Moderate | The RCT design of the intervention strengthens the quality of evidence. However, the absence of reported confidence intervals warrants downgrading for imprecision. Although the study reports a statistically significant effect on cognitive reappraisal compared with the control group, the effect size is small to moderate and does not fully compensate for the lack of precision. Inconsistency, could not be assessed due to the heterogeneity of interventions evaluating the same outcome.Publication bias, could not be evaluated because of the heterogeneity and limited number of available studies. |
| Cognitive reappraisal | ERQ-R | Critical | Patients | UP-CBT | Medication | Farnoodimehr/N=19/18 (UP-BCT/Medication) | Pre : M=19.11, Post : M=24.05, g=NR | NR/ <.001 | Very low concern | Very low concern | NA | Very low concern | Moderate | NA | Moderate | Although the study design is RCT and there is statistical significance in the improvement of cognitive restructuring, several key data required for a full assessment are missing (e.g., confidence intervals, standardized effect size), which weakens the strength of the recommendation for this outcome. This limitation is further compounded by the relatively small sample size. Inconsistency, could not be assessed due to the heterogeneity of interventions evaluating the same outcome. Publication bias, could not be assessed because of the heterogeneity and limited number of studies available. |
| Cognitive reappraisal | UP-CSQ | Critical | Patients | CanCope Mind | CanCope lifestyle | Smith/N=110/114(Cancope Mind/Cancope Lifestyle) | Between group effect  T0→T4 : β = 1.63, SMD = 0.31 T0→T5 : β = 1.85 ; SMD = 0.38 T2→T3 : β = 1.07 ; SMD= 0.22 | T0→T4 : (CI 95% 0.01–3.38)/ .036  T0→T5 : (CI 95% 0.14–3.59)/ .016 T2→T3 : (CI 95% −0.03–2.23)/ .039 | Moderate concern | Moderate concern | NA | Very low concern | Moderate | NA | Moderate | The sample size is adequate to provide a reasonably robust estimate, and the effect size remains small to moderate when averaging the SMD values (0.22–0.38) between groups. The RCT design further strengthens the quality of the recommendation for this outcome. Inconsistency, could not be assessed due to the heterogeneity of interventions evaluating the same outcome. Publication bias, could not be assessed because of the heterogeneity across studies. |
| Decentring | EQ | Critical | Relatives | ERT-C | Waitlist | O'Toole/N=43/37 (ERT-C,Waitlist) | Intervention - Pre : M=33.9, Post : M=39.1 / Control group - Pre : M=34.3, Post : M=34.2, g=0.93 | NR/<.001 | Very low concern | Moderate concern | NA | Very low concern | High | NA | High | The RCT design together with the large effect size increases the overall quality of evidence for this outcome. The sample size is small but the effect is statistically significant. Inconsistency, cannot be assessed as only one study is available for this outcome. Publication bias, cannot be assessed since only one study contributed to this outcome. |
| Expressive supression | ERQ-S | Critical | Patients | UP-BCT | Medication | Farnoodimehr/N=19/18 (UP-BCT/Medication) | Intervention - Pre : M=31.38, Post : M=17.38,g=NR | NR/ <.001 | Very low concern | Very low concern | NA | Very low concern | High | NA | Moderate | Large reduction in expressive suppression observed from pre- to post-intervention (p < .001); although the standardized effect size was not reported, the magnitude suggests a large effect. The RCT design strengthens the quality of evidence. However, the small sample size increases imprecision, and no confidence intervals were reported. Only one study was available for this outcome; therefore, inconsistency could not be assessed. Publication bias could not be evaluated because only one study contributed to this outcome. |
| Belief about Emotion | BES | Critical | Patients | CanCope Mind | CanCope lifestyle | Smith/N=110/114(Cancope Mind/Cancope Lifestyle) | Between group effect  T0→T4 : β = - 5.35, SMD = 0.34 T0→T5 : β = -6.12 ; SMD = .31 T2→T3 : β = 3.58 ; SMD= .25 | T0→T4 : (CI 95% -9.56, - 1.12)/.005 T0→T5 : (CI 95% -11.28, -.90)/ .009  T2→T3 : (CI 95% .57,6.58)/.008 | Moderate concern | Moderate concern | NA | Very low concern | Moderate | NA | Moderate | Small to moderate effects. Some time points show imprecision (CIs close to 0), although the RCT design of the study strengthens the confidence in this intervention for targeting beliefs about emotions. Publication bias could not be assessed because only one study contributed to this outcome. |
| Catastrophizing | CERQ-C | Critical | Patients | CanCope Mind | CanCope lifestyle | Smith/N=110/114(Cancope Mind/Cancope Lifestyle) | Between group effect  T0→T4 : β = - .69 , SMD = .29 T0→T5 : β = -.71 ; SMD = .26 T2→T3 : β = -.06 ; SMD= .02 | T0→T4 : (CI95% -1.47,.02)/ .049 T0→T5 : (CI95% -1.67, .25)/.109 T2→T3 : (CI95% -.36,.29)/.368 | Moderate concern | High concern | NA | Very low concern | Low | NA | Low | Small but inconsistent between-group effects; CIs for T0→T5 and T2→T3 include 0 → downgraded for imprecision. Effect size small (SMD 0.02–0.29). Only one study is available, so it is not possible to assess publication bias. |
| Refocus on planning | CERQ-R | Critical | Patients | CanCope Mind | CanCope lifestyle | Smith/N=110/114(Cancope Mind/Cancope Lifestyle) | Between group effect  T0→T4 : β = .69 , SMD = .19 T0→T5 : β = .50 ; SMD = .15 T2→T3 : β = .08 ; SMD= .02 | T0→T4 : (CI 95% -.51, 1.89)/ .195 T0→T5 : (CI 95% -.73,1.73)/ .358 T2→T3 : (CI 95%-.87,1.03)/ .844 | Moderate concern | High concern | NA | Very low concern | Low | NA | Low | Large confidence intervals include the null effect, indicating imprecision despite a moderate sample size. Effect size is very small, suggesting minimal clinical impact. Publication bias could not be assessed, as only one study contributed to this outcome. |
| Experiential avoidance | MEAQ-30 | Critical | Patients | CanCope Mind | CanCope lifestyle | Smith/N=110/114(Cancope Mind/Cancope Lifestyle) | Between group effect  T0→T4 : β = -8.58 , SMD = .42  T0→T5 : β = -13.89 ; SMD = .69 T2→T3 : β = -1.56 ; SMD= .08 | T0→T4 : (CI95% -14.05, -3.05)/ <.001 T0→T5 : (CI95% -20.59, -7.19)/ <.001  T2→T3 : (CI95% -5.30,2.21)/ .344 | Moderate concern | Moderate concern | NA | Very low concern | Moderate | NA | Moderate | The between-group estimates are statistically significant and show a small-to-moderate effect (SMD 0.42–0.69). However, the intermediate estimate (T2→T3) is not statistically significant (CI includes 0), which lowers our overall confidence. We downgraded the certainty due to this point-specific variation in effect magnitude. Publication bias could not be assessed, as only one study contributed to this outcome. |
| Psychological flexibility | AAQ-II | Critical | Patients | UP-BCT | Medication | Farnoodimehr/N=19/18 (UP-BCT/Medication) | Intervention - Pre : M=30.05, Post : M=22.11, g= NR | NR/ <.001 | Very low concern | High concern | NA | Very low concern | Moderate | NA | Moderate | The estimate is based on a single study. The absence of confidence intervals and standard deviations prevents assessment of precision and calculation of a standardized effect size. The raw mean difference (−8.39) is statistically significant and suggests a clinically meaningful improvement; however, due to the small sample size and lack of confidence intervals, we downgraded the quality of the recommendation. Publication bias could not be assessed, as only one study contributed to this outcome. |
| Psychological distress | CORE-OM | Important | Patients | EFT | Pre-post intervention | Almeida/N=17 | Intervention - Pre : M=18.38, Post : M=13.56, d=0.88 | NR/<.001 | Moderate concern | Moderate concern | NA | Very low concern | High | NA | Moderate | The evidence is based on a single pre-post study. The raw difference between the two time points is −4.82, with a large standardized effect size (d = 0.88) and a statistically significant improvement (p < .001). However, the absence of a control group and the small sample size, along with no reported confidence intervals, lower our certainty in the estimate. |
| Psychological distress | HADS total | Important | Relatives | ERT-C | Pre-post intervention | Applebaum/N=22 | Intervention - Pre : 18.87 / Control group : 14.74, g=0.65 | Difference Pre-Post : (CI95% -4.58, IC95% -7.62 ; -1.54) | Moderate concern | Moderate concern | NA | Very low concern | Moderate | NA | Low | The pre-post comparison shows a statistically significant and clinically meaningful improvement. The availability of the change confidence interval reduces concerns about imprecision. However, because the evidence derives from a single-arm pre-post design (susceptible to confounding and other biases), we downgraded the certainty of evidence for study limitations. Publication bias could not be assessed due to heterogeneity among the studies. |
| Psychological distress | HADS total | Important | Caregivers | ERT-C | Waitlist | O'Toole/N=43/37 | Intervention - Pre : M=20.5, Post : 13.1 / Control group - Pre : M=19.2, Post : M=17.4, g= 0.86 | NR/<.001 | Very low concern | Moderate concern | NA | Very low concern | Moderate | NA | Moderate | The RCT pre-post design increases the quality of the study. Results show a significant reduction in psychological distress with ERT-C. The lack of reported confidence intervals and the small sample size contribute to moderate imprecision. Inconsistency and publication bias cannot be assessed. This evaluation focuses only on caregivers (while HADS total was also assessed in patients), as patients did not receive the intervention. |
| Fear of cancer recurrence | PQ-FCR | Important | Patients | EFT | Pre-post intervention | Almeida/N=17 | Intervention - Pre : M=6.15, Post : M=4.89, d=0.98 | NR/.001 | Moderate | High concern | NA | Very low concern | High | NA | Low | The pre-post study with a small sample downgrades the quality of the evidence for this recommendation. The effect size is large, but imprecision is high due to the small sample and the absence of confidence intervals. Only one study contributes to this outcome, so inconsistency and publication bias cannot be assessed. |
| Rumination | RRS | Important | Relatives | ERT-C | Pre-post intervention | Applebaum/N=22 | Intervention - Pre : M= 45.74, Post : M=42.68, g=0.36 | Pre : (CI 95% 41.65,49.83), Post (CI 95% 39.58,45.79)/.006 | Moderate | Moderate concern | NA | Very low concern | Moderate | NA | Moderate | A pre-post study with 22 participants shows a small-to-moderate reduction in the outcome. Precision is limited by the small sample size, although 95% confidence intervals are reported. Only one study contributes to this outcome, so inconsistency and publication bias cannot be assessed. |
| Rumination | RRS(-B) | Important | Relatives | ERT-C | Waitlist | O'Toole//N=43/37 (ERT-C/Waitlist) | Intervention - Pre : M=10.8, Post : M= 8.7 / Control group - Pre : M= 11., Post : M=10.3, g= 0.24 | NR/.220 | Very low concern | High concern | NA | Very low concern | Low | NA | Low | This RCT shows a small, non-significant effect on the outcome. Imprecision is high due to the small sample size and the absence of confidence intervals. Inconsistency and publication bias could not be assessed for this outcome, as only a single study contributes to the evidence. Therefore, evaluation of heterogeneity between studies and potential publication bias is not possible. |
| Worry | PSWQ | Important | Relatives | ERT-C | Pre-post intervention | Applebaum/N=22 | Intervention - Pre : M=54.77, Post : M=48.68, g=0.47 | NR/<.001 | Moderate | Moderate concern | NA | Very low concern | Moderate | NA | Low | Pre-post design with a small sample. Effect size is moderate (g = 0.47). Precision is limited due to the small sample size and lack of confidence intervals. Inconsistency and publication bias could not be evaluated. |
| Worry | PSWQ | Important | Relatives | ERT-C | Waitlist | O'Toole/N=43/37 (ERT-C/Waitlist) | Intervention - Pre :M=50.9 , Post :M=40.8/ Control - Pre :M=52.6, Post :M=51.4 g= 0.96 | NR/<.001 | Very low concern | Moderate concern | NA | Very low concern | High | NA | Moderate | The study shows a large effect size on worry. The sample size is moderate, but no confidence intervals were reported. Risk of bias is moderate. Inconsistency and publication bias could not be evaluated, as only a single study contributes to this outcome. |
| Caregiver burden | CRA | Important | Relatives | ERT-C | Pre-post intervention | Applebaum/N=22 | Intervention - Pre :M=79.65, Post :M=78.35, g=0.15 | Pre : (CI 95% 76.4,82.89 ), Post (CI 95% 75.08,81.62)/.531 | Moderate | High concern | NA | Very low concern | Low | NA | Very low | Pre-post study showing a very small, non-significant effect. The sample size is small, leading to high imprecision. Risk of bias is moderate, and the certainty of evidence is rated very low. |
| Caregiver burden | CRA | Important | Relatives | ERT-C | Waitlist | O'Toole/N=43/37 (ERT-C/Waitlist) | Intervention - Pre : M=35.7 , Post : M=29.9 / Control group - Pre : M=35.0, Post : M=34.3, g=0.55 | NR/.006 | Very low concern | Moderate concern | NA | Very low concern | Moderate | NA | Moderate | The effect observed in the intervention group is moderate. The sample size is modest, and no confidence intervals are reported. Overall confidence in this effect is rated as moderate. |
| Depression | HADS-d | Important | Patients and Relatives | ERT-C | Pre-post intervention | Applebaum/N=22 | Intervention - Pre :M= 7.32, Post : M=5.37, g= 0.49 | Pre : (CI 95% 10.36,12.74), Post (CI 95% 7.98,10.76)/.005 | Moderate | High concern | NA | Very low concern | Moderate | NA | Low | Pre-post study with a small sample, showing a moderate effect, precision is limited due to the small sample size. |
| Depression | CES-D, | Important | Patients | PSMT | Usual care | Jacobsen/N=125/132 (PSMT/UC) | Comparison of UC with PSMT : F=2.51 | NR/Comparison of UC with PSMT : .11 | Very low concern | Moderate concern | NA | Very low concern | Low | NA | Low | Effect not statistically significant, no confidence intervals reported, effect size small, overall confidence rated as low. |
| Depression | CES-D, | Important | Patients | SSMT | Usual care | Jacobsen/N=125/132 (SSMT/UC) | Comparison of UC with SSMT : F=7.01 | NR/Comparison of UC with SSMT :.009 | Very low concern | Moderate concern | NA | Very low concern | Moderate | NA | Moderate | Effect statistically significant, no confidence intervals reported, effect size small to moderate, overall confidence rated as moderate. |
| Depression | BSI-d | Important | Patients | CBSM | Usual care | Nápoles/N=76/75 (CBSM/UC) | Intervention - Pre : M=0.93 , Post : 0.38/ Control group,- Pre : M=0.75, Post : 0.46 g=NR | NR/Pre:.164, Post:.355 (group mean) | Very low concern | High concern | NA | Very low concern | Low | NA | Low | No significant between-group differences were observed for the outcome. Reported p-values were non-significant, and no confidence intervals or standardized effect sizes were provided. The combination of limited precision and absence of a meaningful effect leads to a downgrade for imprecision (high concern) and indicates a low effect size. Overall, the certainty of evidence for this outcome is rated as low. |
| Depression | BSI-d | Important | Patients | BCI | Usual care | Thilges/N=12/12(BCI/UC) | Intervention - Pre :M= 49.44, Post :43.57, Control group - Pre : M=50.85, Post: 52.23, g=NR | NR/NR | Very low concern | High concern | NA | Very low concern | NR | NA | Low | The data show a mean decrease after the intervention, whereas the control group slightly worsened. However, the absence of confidence intervals, p-values, and standardized effect size estimates, combined with the very small total sample, results in substantial imprecision. |
| Depression | DASS-d | Important | Patients | UP-CBT | Medication | Farnoodimehr/N=19/18 (UP-BCT/Medication) | Between group : F=49.36, partial eta : 0.50 | NR/0.01 | Very low concern | Low | NA | Very low concern | High | NA | High | UP-CBT intervention showed a significant reduction in depression compared to the control group. The effect size is large, suggesting a substantial impact. Although no confidence interval is reported, limiting full assessment of precision, the statistically significant result and reasonable sample size support moderate-to-high confidence in this estimate. |
| Anxiety | HADS-a | Important | Caregivers | ERT-C | Pre-post intervention | Applebaum/N=22 | Intervention - Pre : M=11.55, Post :M=9.37, g=0.66 | Pre : (CI95% 10.36,12.74), Post : (IC95% 7.98,10.76)/.005 | Very low concern | Moderate concern | NA | Very low concern | Moderate | NA | Moderate | The pre-post study shows a significant decrease in the outcome. However, the absence of a control group limits confidence in the estimated effect despite statistical significance (p = 0.005). The moderately wide confidence interval justifies downgrading for imprecision. |
| Anxiety | BSI-a | Important | Patients | BCI | Usual care | Thilges/N=12/12(BCI/UC) | Intervention - Pre :M=44.20 , Post :M= 42.11, Control group - Pre :M= 44.20, Post :M= 47.98, g=NR | NR/NR | Very low concern | High concern | NA | Very low concern | NR | NA | Low | The data indicate a decrease in the outcome for the intervention group, while the control group slightly worsened. However, the absence of reported effect size, confidence intervals, and p-values, along with the very small sample size, results in high imprecision and limits confidence in the effect estimate. |
| Anxiety | BSI-a | Important | Patients | CBSM | Usual care | Nápoles/N=76/75 (CBSM/UC) | Intervention - Pre : M=0.93, Post :0.39/ Control group - Pre : M=1.01, Post : M=0.58, g=NR | NR/pre:.577, post:.465 | Very low concern | High concern | NA | Very low concern | Low | NA | Low | The intervention group showed a slight decrease in anxiety compared to the control group, but the between-group difference was not statistically significant. Due to the small magnitude change and lack of significance, the effect on anxiety is considered minimal and uncertain. |
| Anxiety | STAI-S | Important | Patients | PSMT and SSMT | Usual care | Jacobsen/N=125/132 (PSMT/UC) | Comparison of UC with PSMT : F=0.11 | NR/Comparison of UC with PSMT : .74 | Very low concern | High concern | NA | Very low concern | Low | NA | Low | The between-group effect was very small (F = 0.11) and not statistically significant (p = 0.74). No confidence intervals or standardized effect sizes were reported. Imprecision is high because of the absence of CIs and a non-significant effect. |
| Anxiety | STAI-S | Important | Patients | PSMT and SSMT | Usual care | Jacobsen/N=125/132 (SSMT/UC) | Comparison of UC with SSMT : F=5.18 | NR/Comparison of UC with SSMT:.02 | Very low concern | Moderate concern | NA | Very low concern | Low | NA | Low | The comparison of UC with SSMT shows a statistically significant difference (F = 5.18, p = 0.02); however, no standardized effect size (SMD or g) or confidence interval is reported. Therefore, while an effect is likely present, its magnitude is uncertain and is considered probably small. Imprecision is high due to the lack of these estimates, and the small sample size further limits confidence in the result |
| Anxiety | DASS-a | Important | Patients | UP-CBT | Medication | Farnoodimehr/N=19/18 (UP-BCT/Medication) | Between group : F=312.80, partial eta : 0.62 | NR/0.01 | Very low concern | Moderate concern | NA | Very low concern | High | NA | Moderate | The between-group analysis for DASS-A shows a very large effect (partial η² = 0.62) and statistically significant differences (F = 312.80, p = 0.01). Despite the very large effect size, the small sample size reduces precision. Inconsistency cannot be evaluated with a single study. |
| Stress | DASS-s | Important | Patients | UP-CBT | Medication | Farnoodimehr/N=19/18 (UP-BCT/Medication) | Between group : F=72.33, partial eta : 0.67 | NR/0.01 | Very low concern | Moderate concern | NA | Very low concern | High | NA | Moderate | The between-group effect shows a very large effect size (partial eta = 0.67). However, the small sample size (N = 19 intervention, N = 18 control) and potential methodological limitations reduce our confidence in the estimate. Overall, the quality of evidence is rated as moderate |
| Quality of life | FACT-H&N | Important | Patients | BCI | Usual care | Thilges/N=12/12(BCI/UC) | Intervention - Pre :M=19.40 , Post :20.60, g=NR | NR/NR | Very low concern | Moderate concern | NA | Very low concern | High | NA | Low | The data show a small increase in the outcome from pre- to post-intervention in the intervention group compared with the control group. However, no standardized effect size, confidence interval, or p-value is reported, and the sample size is very small (N = 12 per group), resulting in substantial imprecision and uncertainty regarding the effect. Risk of bias cannot be fully assessed due to missing methodological details |
| Quality of life | SF-36 | Important | Patients | PSMT | Usual care | Jacobsen/N=125/132 (PSMT/UC) | Comparison of UC with PSMT : F=0.03 | NR/Comparison with PSMT : .87 | Very low concern | High concern | NA | Very low concern | Low | NA | Low | The comparison between UC and PSMT shows a negligible effect (F = 0.03, p = .87) with no CI reported. The lack of precision and very small observed effect reduce confidence in the estimate |
| Quality of life | SF-37 | Important | Patients | SSMT | Usual care | Jacobsen/N=125/132 (SSMT/UC) | Comparison of UC with SSMT : F=0.37 | NR/Comparison with SSMT : .54 | Very low concern | High concern | NA | Very low concern | Low | NA | Low | The comparison between UC and SSMT shows a very small, non-significant effect (F = 0.37, p = .54) with no CI reported. The lack of precision and minimal observed effect reduce confidence in the estimate. |
| Quality of life | FACT-B | Important | Patients | CBSM | Usual care | Nápoles/N=76/75 (CBSM/UC) | Intervention - Pre :M=66.46, Post :M=80.64/ Control group - Pre : M=68.83, Post : 77.02 , g=NR | NR/pre:.37, post:.174 | Very low concern | High concern | NA | Very low concern | Low | NA | Low | The CBSM intervention compared to UC shows minimal improvement in scores, with non-significant p-values (pre p = .37, post p = .174) and no reported effect size or CI. The small sample, lack of precision, and limited effect reduce confidence in the estimate. |
| Quality of life | EORCT-QLQ-C30 | Important | Patients | ERT-C | Waitlist | O'Toole/N=43/37 (ERT-C/Waitlist) | Intervention - Pre :M=53.4 , Post :M=67.2/Control group - Pre : M=57.2, Post : M=54.5, g=0.88 | NR/.019 | Very low concern | Moderate concern | NA | Very low concern | High | NA | Moderate | Rhe ERT-C intervention shows a large improvement in scores compared to the waitlist control , with a large effect size (g = 0.88) and p = .019. he modest sample size and absence of reported confidence intervals reduce the precision of the estimate. |
| Quality of life | WHO-5 | Important | Relatives | ERT-C | Waitlist | O'Toole/N=43/37 (ERT-C/Waitlist) | Intervention - Pre :9.2 , Post :15.5, / Control group - Pre : M=10.2, Post : M=12.3, g=0.79 | NR/.001 | Very low concern | Moderate concern | NA | Very low concern | High | NA | Moderate | The intervention shows a substantial improvement compared to the control group, with a large effect size (g = 0.79, p = .001). Precision is limited due to the small sample size and lack of confidence intervals |

**Legend*:*** Questionnaires: *CBI (Cognitive Behavioral Intervention), EFT-CA (Emotional Freedom Technique for Caregivers), ERT-C (Emotional Regulation Training for Caregivers), UP (Unified Protocol), AAQ-II (Acceptance and Action Questionnaire), BDI-II (Beck Depression Inventory-II), BES (Beliefs about Emotion Scale), BSI (Brief Symptom Inventory), BSI-18 (Brief Symptom Inventory-18), CaBI (Cancer Behavioral Inventory-Brief Form), Caregiver Reaction Assessment (CRA), CERQ-C (Catastrophizing), CERQ-R (Refocus on Planning), CES-D (Center for Epidemiologic Studies Depression Scale), CORE-OM (Clinical Outcome in Routine Evaluation), DASS (Depression, Anxiety, Stress Scale), DERS (Difficulties in Emotion Regulation Scale), DT (Distress Thermometer), EQ (Experiences Questionnaire), FACT-B (Functional Assessment of Cancer Therapy-Breast), FACT-H&N (Functional Assessment of Cancer Therapy-Head and Neck Version), FFMQ (Five Facet Mindfulness Questionnaire), HADS (Hospital Anxiety Depression Scale), IES (Intrusive Events Scale), ITS (Intrusive Thoughts Scale), MEAQ-30 (Multidimensional Experiential Avoidance Questionnaire-30), PQ (Personal Questionnaire), PSWQ (Penn State Worry Questionnaire), QLQ-C30 (Cancer Core Quality of Life Questionnaire), RRS (Rumination Response Scale), RRS-B (Rumination Response Scale – Brooding subscale), SF-36 (Medical Outcomes Study 36-Item Short Form), SMQ (Southampton Mindfulness Questionnaire), STAI-S (State-Trait Anxiety Inventory Scale), UP-CSQ (Unified Protocol-Cognitive Skills Questionnaire), WHO-5 (World Health Organization Questionnaire).*

*Interventions: CBI : Cognitive Behavioral Intervention, CM : CanCopeMind, EFT-CA: Emotion Focused Therapy-Caregiver, ERT-C : Emotional Regulation Training-Caregivers , UP-CBT : Unified Protocol- Cognitive Behavioral Therapy*

*Other abbreviations:* PSMT = Professionally administered stress management training; SSMT = Self-administered stress management training, NA = Not Applicable, NR = Not Reported
