# Supplementary material for: Emotion Regulation Interventions for Cancer Patients and Their Relatives: A Systematic Review
Source: Cancer Med. 2026 Feb 12;15(2):e71514. doi: 10.1002/cam4.71514 (PMC12900081; doi:10.1002/cam4.71514)
Supplement: Supplementary file 2 — Appendix B Review. [file CAM4-15-e71514-s002.docx]

review_380249_20250922015350

| **Reviewer A** | **Reviewer B** | **A Yes, B Yes** | **A Yes, B No** | **A No, B Yes** | **A No, B No** | **Proportionate Agreemen** | **Yes Probabilit** | **No Probability** | **Random Agreement Probabilit** | **Cohen's Kappa** |
| --- | --- | --- | --- | --- | --- | --- | --- | --- | --- | --- |
| **Ambre Naeyaert** | Kristopher Lamore | 2 | 8 | 2 | 6751 | 0.99852 | 0.0 | 0.99793 | 0.99793 | 0.28511 |
| **Ambre Naeyaert** | Valentyn Fournier | 14 | 105 | 68 | 20732 | 0.99173 | 0.00002 | 0.99041 | 0.99044 | 0.13529 |
| **Kristopher Lamore** | Valentyn Fournier | 3 | 5 | 25 | 3363 | 0.99117 | 0.00002 | 0.98942 | 0.98944 | 0.1636 |
| **Kristopher Lamore** | Marie-Charlotte Gandolphe | 0 | 0 | 0 | 633 | 1.0 | 0.0 | 1.0 | 1.0 | NaN |
| **Ambre Naeyaert** | Marie Vander Haegen | 0 | 3 | 15 | 327 | 0.94783 | 0.00038 | 0.9482 | 0.94858 | -0.01471 |
| **Ambre Naeyaert** | Marie-Charlotte Gandolphe | 0 | 0 | 1 | 1723 | 0.99942 | 0.0 | 0.99942 | 0.99942 | 0.0 |
| **Marie-Charlotte Gandolph** | Valentyn Fournier | 1 | 1 | 0 | 293 | 0.99661 | 0.00002 | 0.98985 | 0.98988 | 0.66515 |
| **Kristopher Lamore** | Marie Vander Haegen | 0 | 0 | 0 | 72 | 1.0 | 0.0 | 1.0 | 1.0 | NaN |
| **Marie Vander Haegen** | Valentyn Fournier | 9 | 31 | 16 | 350 | 0.88424 | 0.00607 | 0.84597 | 0.85203 | 0.21763 |
| **Marie Vander Haegen** | Marie-Charlotte Gandolphe | 1 | 7 | 3 | 574 | 0.98291 | 0.00009 | 0.97958 | 0.97967 | 0.159 |
| **Laura Caton** | Valentyn Fournier | 2 | 7 | 0 | 133 | 0.9507 | 0.00089 | 0.92343 | 0.92432 | 0.34862 |
| **Ambre Naeyaert** | Laura Caton | 3 | 1 | 18 | 961 | 0.98067 | 0.00009 | 0.97465 | 0.97474 | 0.23477 |
| **Kristopher Lamore** | Laura Caton | 0 | 0 | 4 | 585 | 0.99321 | 0.0 | 0.99321 | 0.99321 | 0.0 |

1
